# Heat-stress-induced sprouting and differential gene expression in growing potato tubers: Comparative transcriptomics with that induced by postharvest sprouting

**DOI:** 10.1038/s41438-021-00680-2

**Published:** 2021-10-15

**Authors:** Guodong Zhang, Ruimin Tang, Suyan Niu, Huaijun Si, Qing Yang, Om P. Rajora, Xiu-Qing Li

**Affiliations:** 1 Gansu Provincial Key Laboratory of Aridland Crop Science, Gansu Key Laboratory of Crop Genetic and Germplasm Enhancement, Gansu Agricultural University, Lanzhou, China; 2 College of Agronomy, Gansu Agricultural University, Lanzhou, China; 3 Fredericton Research and Development Centre, Agriculture and Agri-Food Canada, Government of Canada, Fredericton, New Brunswick Canada; 4 College of Life Sciences, Nanjing Agricultural University, Nanjing, China; 5 Institute of Bioengineering, Zhengzhou Normal University, Zhengzhou, China; 6 College of Life Science and Technology, Gansu Agricultural University, Lanzhou, China; 7 Faculty of Forestry and Environmental Management, University of New Brunswick, Fredericton, Canada

**Keywords:** Transcriptomics, Plant sciences, Plant development

## Abstract

Crops face increased risk from heat stress due to climate change. Potato (*Solanum tuberosum* L.) tubers grown in hot summers often have defects including pre-harvest sprouting (“heat sprouts”). We have used 18 potato cultivars to investigate whether heat stress (HS) conditions alone could cause heat sprouting and dormancy changes in tubers. We also examined transcriptomic responses of potato to HS and whether these responses are like those induced by postharvest sprouting. We demonstrated that HS alone caused heat sprouts and shortened postharvest dormancy period, heat-sprouted tubers became dormant after harvest, and cultivars varied substantially for producing heat spouts but there was no clear association with cultivar maturity earliness. Cultivar Innovator did not show any heat sprouts and still had long dormancy. Dormancy-associated genes (*DOG1* and *SLP*) were downregulated in HS tubers like in postharvest sprouting tubers. We have identified 1201 differentially expressed genes, 14 enriched GO terms and 12 enriched KEGG pathways in response to HS in growing tubers of ‘Russet Burbank’. Transcriptomic response of ‘Russet Burbank’ to HS showed significant similarities to that of postharvest non-HS sprouted tubers. Gibberellin biosynthesis pathway was enriched in heat-stressed tubers and was likely involved in heat sprouting and dormancy release. Heat sprouting and postharvest sprouting shared common candidate genes and had significant similarity in gene expression. Our study has significance for selecting potato cultivars for farming, planning storage and utilization of heat-stressed tubers, identifying sprouting-related genes, understanding heat-stress biology, and breeding heat-tolerant potato cultivars, especially for sustainable potato production under climate change.

## Introduction

Potato (*Solanum tuberosum* L.), one of the most important staple crops worldwide, is very sensitive to heat stress^1,2^. Immature tubers often sprout during growth if potato plants are grown in hot seasons^3^, a phenomenon called “heat sprouts”^4^ or “heat sprouting”^5^. More than a half of tubers in the field could be sprouted^6^. The quality and usability of potatoes are severely reduced if potato tubers sprout before harvest or shortly after harvest. Because storability of potatoes is critical for their usability and the year-round supply, heat-induced sprouts and postharvest dormancy shortening can negatively affect potato agriculture. Potato agriculture will likely face increased global warming with climate change. Therefore, it is critical to understand the effects of heat stress (HS) alone on potato tuber sprouting and associated gene regulation.

Heat stress on field potato crops can also shorten or abolish the dormancy of potato tubers;^7^ whereas the non-stressed potato tubers normally sprout only after a few months of postharvest storage^8^. Dormancy length or storability is regulated by several factors, including environment, tuber physiology, and the expression of genes involved^9^. Potato industry usually stores harvested potatoes under low temperatures to delay sprouting^10^.

Studies on heat sprouting often have been conducted by comparing the potato crop planted in the spring and the crop planted in summer^4,11^ because temperature in the field cannot be easily controlled. Another approach is to grow potato plants in pots in different months of the year, and consider plants grown in the hot season as heat treatment and plants grown in the optimal season as the control^12^. The advantage of these studies is that potato plants receive heat stress by natural day/night temperatures in the hot summer, and the field soil is less hot than the air, but the disadvantage is the lack of the exact control and has confounding effects of other factors. Temperature is not the only different factor between the potato plants growing in the spring and summer seasons. There are also differences in day length, soil moisture, atmospheric humidity, light intensity, diseases, and other conditions. It is not surprising that the results are somewhat different, even reversed between years^11^ or between different studies. For example, about 57% of tubers of the cultivar ‘Russet Burbank’ had heat sprouts when grown under drought in the summer of 1985 but no heat sprouts in the summer of 1986 for potatoes planted in June (much less rainfall for June in 1985 than in 1986)^6^. Therefore, it appears that low soil moisture may play an important role in producing heat sprouts too. However, drought in the field was found to shorten seed potato dormancy^13^, but did not induce heat sprouts in a study of potted plants^12^. Studies on heat spouting by comparing spring potato plants with summer potato plants cannot establish whether the heat sprouts are due to only heat stress or combined effects of both heat stress and water deficiency stress. In such cases, since one cannot be absolutely sure which factors caused “heat sprouts”, sometimes “heat sprouts” are also called “immature tuber sprouts”^6^ or more safely “field sprouts”^2^.

The effect of heat stress on sprouting has also been tested by growing potato plants in potted quartz sand in growth chambers^14^. In that study, the duration of dormancy period of postharvest heat-stressed tubers was shortened in an early cultivar Diamant but not shortened in a medium-late cultivar Désirée when plants were treated with high temperatures (30 °C/24 °C for day/night temperatures) after tuber initiation (65 days after planting)^14^. To facilitate testing the effect of different nitrogen fertilization levels, plants in that experiment were grown in pure quartz sand. The authors observed that quartz sand did not hold water well during the days of high temperature treatments (30 °C/24 °C for day/night temperatures) and some plants were partially wilted and suffered certain levels of water deficiency stress^14^. Thus, in that study also, the effect of heat stress on tuber sprouting was confounded with that of water deficiency stress. Therefore, experiments with specially-designed potting mix and sensor-controlled air humidity to ensure no drought stress during heat stress treatment are required to further investigate whether heat stress alone without water stress can effectively induce heat sprouts.

Potato cultivars may differ in their response to produce heat sprouts and for dormancy duration. However, to the best of our knowledge, all studies examining the effect of heat on potato tuber sprouting and the length of dormancy were conducted using one or a few cultivars. Therefore, there is a great need to test a larger number of potato cultivars to investigate inter-cultivar variation for the production of heat sprouts and the duration of postharvest dormancy.

The transition from postharvest dormancy to post-dormancy sprouting potentially involves several physiological processes and differential regulation of many genes. Liu et al.^15^ used reverse-transcriptase quantitative real-time polymerase chain reaction (RT-qPCR) to analyze postharvest non-stressed potato tubers and identified two dormancy-associated genes (*DOG1*, delay of germination 1; *SLP*, a protease gene) that were highly expressed during dormancy and downregulated during dormancy release. At least one sprouting-associated gene, namely *CYP707A1* (coding for cytochrome P450, family 707, subfamily A, polypeptide 1) with increased expression during sprouting, was also identified in the same study^15^. However, it is unclear whether these dormancy- or sprout-associated (or related) genes express in the same way in the heat sprouted or heat stressed tubers as in postharvest sprouting tubers.

High-throughput mRNA sequencing (RNA-Seq) has been used for gene expression analysis of postharvest dormant and post-dormancy normally-sprouted tubers of the cultivar Russet Burbank^16^ and some other cultivars^17,18^ and characterization of the activities of specific transcription factors^19^. A large number of genes (2132) were found to be differentially expressed in dormant non-sprouted versus post-dormancy sprouting tubers of the cultivar Russet Burbank^16^. However, it is unknown whether and to which extent immature heat-stressed tubers share the same response for differentially expressed genes (DEGs), biological processes and metabolic pathways with that of postharvest sprouted tubers. Answering this question may help to understand whether the same/similar pathways, processes and genes are involved in both postharvest sprouted tubers and heat-stressed tubers.

The main objectives of this study were (1) to investigate the effect of heat stress alone on sprouting of growing tubers and postharvest dormancy length, and variation among potato cultivars for these traits, and (2) to identify genes and pathways differentially regulated between heat-stressed and non-heat-stressed (control) tubers and whether the differential regulation was similar to that previously reported for normal post-dormant sprouting versus postharvest dormant tubers. We also studied whether heat sprouting is correlated with the earliness of field maturity among cultivars and whether heat-sprouted potatoes continue to sprout after harvest. We hypothesize that there is among-cultivar variation for producing heat sprouts, and heat-stressed and normal post-harvest sprouting share similar regulation of some genes, biological processes, and pathways.

## Results

### Heat sprouts and variation among cultivars

**Fig. 1:**
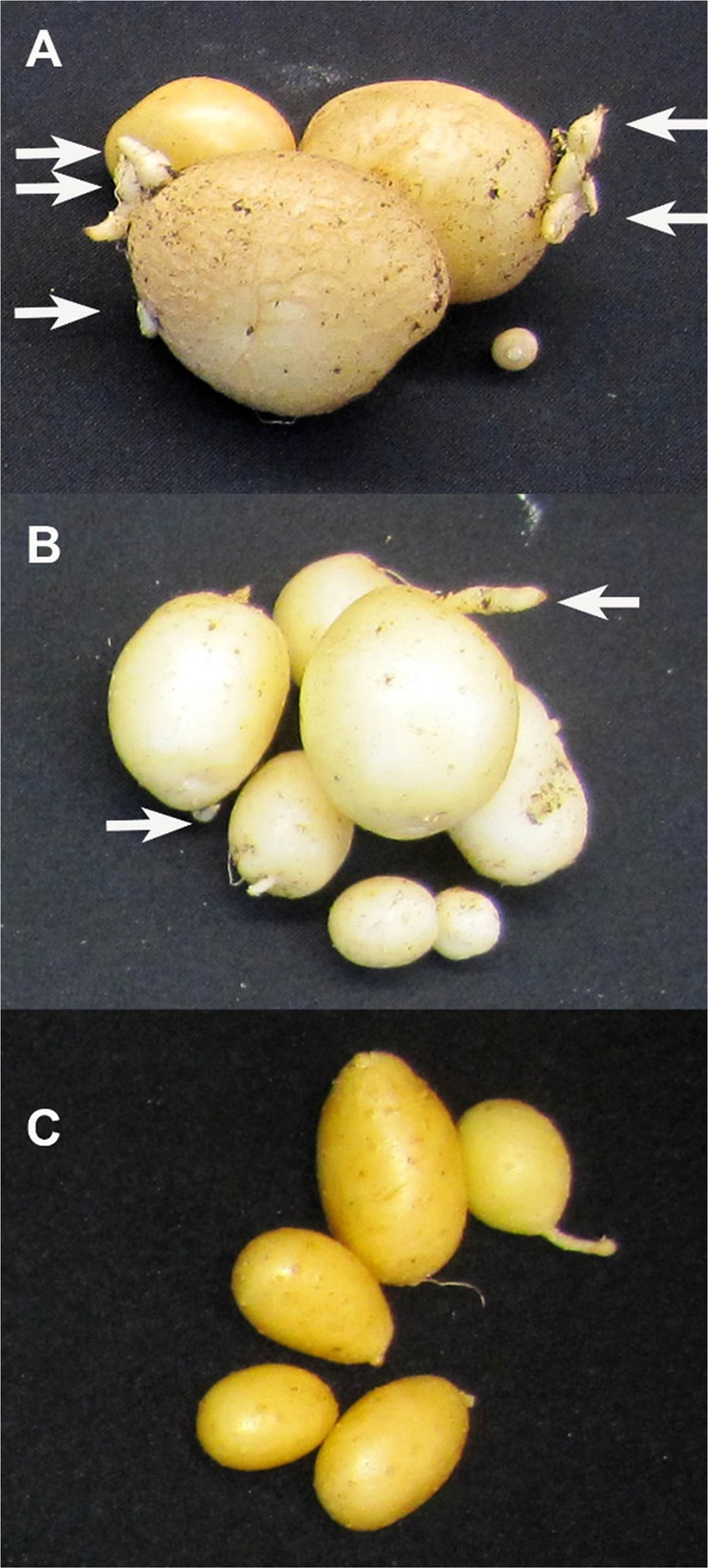
Tubers from heat stressed plants. A: cultivar Eramosa; B: cultivar Nipigon; C: cultivar Innovator. Cultivars Eramosa and Nipigon (**A**, **B**) had heat sprouts, and cultivar Innovator (**C**) had no heat sprouts

The control (CK) plants of all 18 tested cultivars produced 35 to 55 tubers per cultivar, with an average of 44.5 tubers/cultivar and 5.6 tubers/plant, and did not have any heat sprouts. However, the HS plants produced 22.6 tubers per cultivar (2.8 tubers/plant), which was significantly lower than that of the CK plants (*P* < 0.01, two-way ANOVA). The following seven cultivars had heat sprouts: ‘Cherry Red’, ‘Chieftain’, ‘Eramosa’ (Fig. 1A), ‘Goldrush’, ‘Nipigon’ (Fig. 1B), ‘Russet Norkotah’, and ‘Superior’. The other 11 cultivars (such as ‘Innovator’, Fig. 1C) did not have any heat sprouts. Cultivars Atlantic and Shepody produced 17, and 18 tubers, respectively in the HS treatment but none of them had any heat sprout. Cultivar Russet Burbank produced only one tuber in the HS plants and did not have any heat sprout. Cultivar Nipigon produced 21 tubers under heat stress, and 3 of them had heat sprouts, even though very short (Fig. 1B). The average for the longest heat sprout per cultivar for the 18 cultivars was 6.1 cm for the heat treatment group and 0 cm for the control group. Although relatively small number of tubers were used in the test, the 18 cultivars together undoubtedly showed that heat treatment caused heat sprouting because the differences were also statistically significant (*P* < 0.05).

### Heat sprouting in early, late and intermediate maturing cultivars

According to the usual earliness in maturity in the field in North America, the 18 cultivars were classified as early (7 cultivars: Atlantic, Shepody, AC Belmont, Epicure, Eramosa, Innovator, Mirton Pearl, and Superior), late (4 cultivars: Nipigon, Russet Burbank, Denali, and Raritan), or intermediate (6 cultivars: Cherry Red, Chieftain, Goldrush, MaineChip, Red Cloud, Russet Norkotah)^20^. Two (‘Eramosa’ and ‘Superior’) of the eight early maturity cultivars had heat sprouts under HS conditions. Other early cultivars (AC Belmont, Atlantic, Epicure, Innovator, Mirton Pearl, and Shepody) did not have any heat sprouts. Twelve of the 24 tubers of ‘Eramosa’ under heat stress had heat sprouts (Fig. 1A), making ‘Eramosa’ the most susceptible cultivar to heat sprouting among the 18 cultivars tested. Among the six cultivars with intermediate field maturity time, four cultivars (Cherry Red, Chieftain, Goldrush, and Russet Norkotah) had heat sprouts, and only two intermediate maturing cultivars (MaineChip and Red Cloud) did not produce heat sprouts. Among the four late maturing cultivars (Denali, Nipigon, Raritan, and Russet Burbank) under HS conditions, ‘Russet Burbank’ did not produce more than one tuber, which did not produce any heat sprouts. The two other very late maturing cultivars Denali and Raritan did not have any heat sprouts either. Among the late maturing cultivars, only ‘Nipigon’ had some heat sprouts in 3 of 21 tubers from the HS treatment, with the longest sprout of approximately 3 mm in length) (Fig. 1B). Heat sprouts can occur in early, intermediate, and late maturing cultivars and had no significant association with the maturity period class/group among the 18 cultivars tested (*P* > 0.05, Chi-Square test).

### Postharvest dormancy

**Fig. 2:**
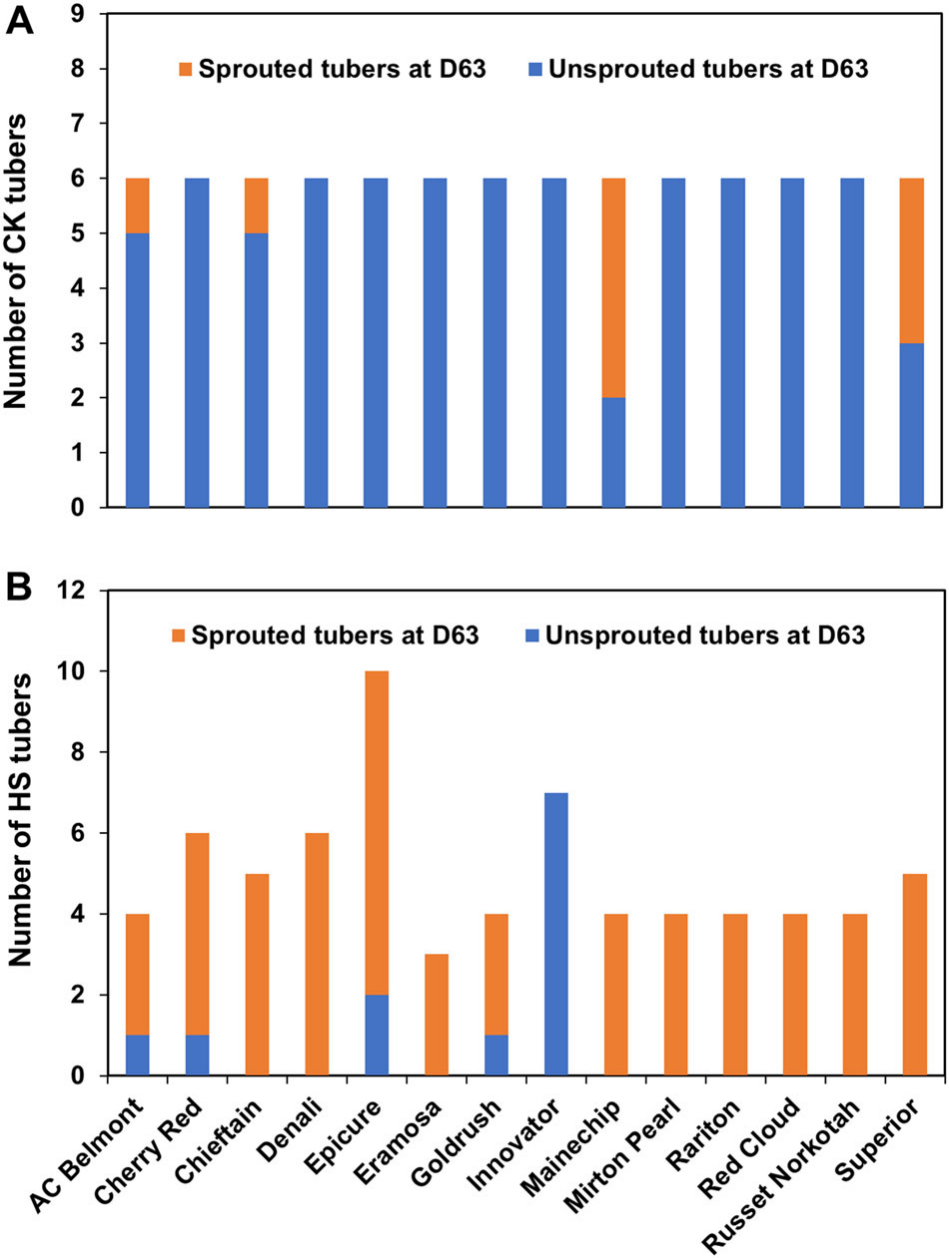
Number of sprouted and non-sprouted tubers of 14 cultivars at Day 63 of storage after harvest from control and heat stressed plants. **A** Control (CK, no heat stress). **B** Tubers from heat stressed plants

Tubers of ‘Atlantic’, ‘Nipigon’, ‘Shepody’, and ‘Russet Burbank’ were used for gene expression analysis; thus, did not have enough tubers for the postharvest storage sprouting test. For the 14 cultivars used in postharvest storage, all tubers became dormant after harvest, regardless of whether they were heat sprouted or not heat sprouted in the soil. The tubers of most cultivars from the HS treatment, except for cultivar Innovator, sprouted on Day 63 of storage while very few tubers sprouted in storage from the control plants (Fig. 2). Among all cultivars, the cultivar Innovator had the longest dormancy for tubers from the HS treatment. On Day 106 in storage, three of the 7 tubers of Innovator from the HS treatment had sprouts while its 6 tubers from control still showed no sprouts. Tubers from the HS plants sprouted earlier than those from the CK treatment during postharvest storage (*P* < 0.01, t-test between HS and CK using sprouted/total ratios).

### RT-ddPCR analysis of the expression patterns of tuber dormancy marker genes in control versus HS tubers

**Fig. 3:**
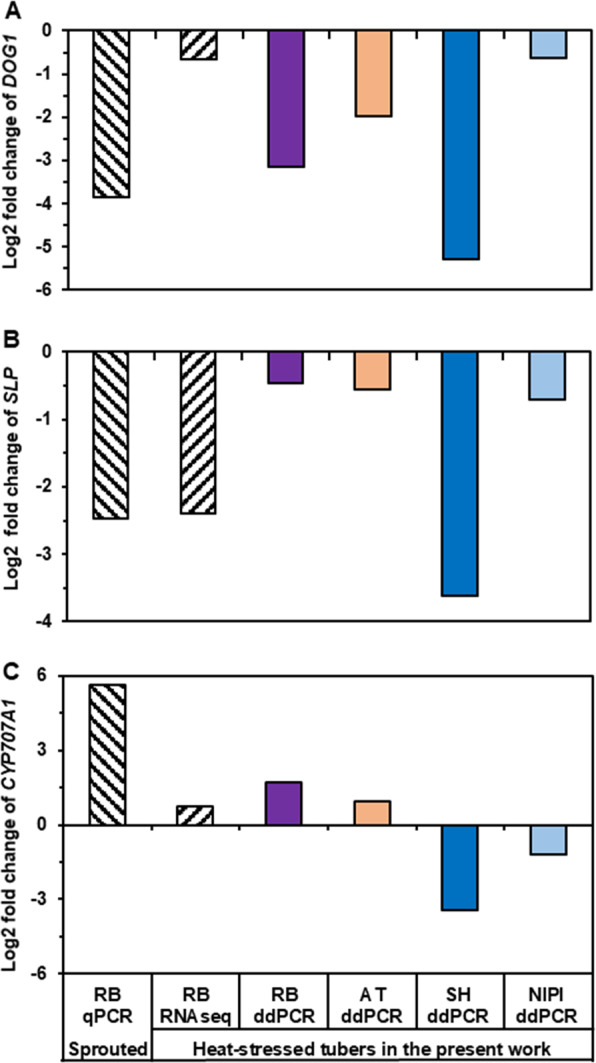
Gene expression change of two dormancy-marker genes (*DOG1* and *SLP*) and one sprouting-marker gene (*CYP707A1*) in heat stressed tubers of potato in comparison with available information in the literature. Log_2_ fold changes are the log_2_ fold changes in genes expression between HS tubers/CK tubers in our present study or between sprouted and non-sprouted postharvest tubers in *Liu 2017*^15^. RB: cultivar Russet Burbank. AT: cultivar Atlantic. SH: cultivar Shepody. NIPI: cultivar Nipigon. PCR: Polymerase chain reaction. qPCR: Reverses transcriptase real-time quantitative PCR. ddPCR: Reverse transcriptase digital droplet PCR. RNA-seq: RNA Illumina sequencing. RB qPCR: data from *Liu 2017*^15^, and all the other data are from our present study

The expression levels of two dormancy-associated genes (*DOG1* and *SLP*^15^) in tubers from heat-stress treatment of all four studied cultivars (Atlantic, Nipigon, Russet Burbank, and Shepody) decreased as compared to the expression levels in tubers of these cultivars from the non-heat stressed control according to the analysis using reverse transcriptase digital droplet PCR (RT-ddPCR) (Fig. 3). The pattern was similar to that observed from RNA-Seq results for these two genes (Fig. 3). Compared to the non-heat-stressed plants (CK) tubers, heat-stressed (HS) plants tubers of all these four cultivars showed decreased expression of these dormancy-associated genes.

### RT-ddPCR analysis of the expression pattern of a tuber sprouting-associated gene in control and HS plant tubers

The RT-ddPCR detected upregulation of the gene *CYP707A1* in tubers of ‘Atlantic’ and ‘Russet Burbank’ from heat stress treatment as compared to the control (Fig. 3C). However, in contrast, ‘Shepody’ and ‘Nipigon’ showed downregulation of this gene in HS plants tubers as compared to the CK control (Fig. 3C). The inconsistency of RT-ddPCR results of the gene *CYP707A1* among these four cultivars suggests that this gene is not a reliable marker for HS-induced dormancy release in potatoes.

### RNA-Seq transcriptome analysis of differentially expressed genes (DEGs) in response to heat stress, and comparative transcriptomics with DEGs in postharvest sprouting tubers

After trimming, cleaning, mapping, and de-duplication using the unimap pipeline of TBSPG^21^, HS and CK plants tubers had 24,628,632 and 23,822,496 Illumina reads, which were mapped uniquely to 26,783 and 25,076 genes (representative transcripts), respectively, to the PGSC_DM_v4.03_transcript-update representative.fasta (http://solanaceae.plantbiology.msu.edu/pgsc_download.shtml).

In total, 1201 genes (Table S2) in the Illumina RNA-Seq transcriptome of ‘Russet Burbank’ showed differentially expression (*P* < 0.05) between HS and CK plant tubers. The fold changes in expression of selected genes from Illumina sequencing was validated by RT-ddPCR (*R*^*2*^ = 0.8958; Fig. S1) using 9 genes and their primers listed in Table S1. Most heat shock proteins (such as PGSC0003DMT400007587) were down regulated. Some invertases (such as PGSC0003DMT400072606), cellulases (PGSC0003DMT400081314), and amylases (PGSC0003DMT400020591) were up regulated when the plants were grown for months under high temperature conditions.

From the heat stress-induced DEGs that we identified, 190 DEGs were the same that were reported as differentially expressed between dormant and sprouting (sprouting/dormant) tubers of the same cultivar Russet Burbank by Campbell et al.^16^ (Table S3). The HS/CK gene expression fold changes in our present study were significantly correlated with the sprouting/dormant gene expression fold changes of non-HS tubers in the previous study^16^ for the common 190 differentially expressed genes (*R* = 0.2, *P* = 6.12E-11) (Table S4). For the 190 differentially expressed genes shared between our study and Campbell et al.^16^ study, the top 30 most differentially expressed genes in terms of *P* values we observed are listed in Table 1. The direction (up- or down- regulation) of gene expression of the top 30 genes sorted by *P* values was largely the same for both our study and previous postharvest sprouting/dormant tuber transcriptomics study^16^ (Table 1).

**Table 1:** Comparison of the up- or down regulation for the 30 most significant differentially expressed genes between our present study and Campbell 2014^16^ study of RNA-Seq of the same cultivar—Russet Burbank

Potato transcript ID	Gene product	This study log_2_ fold change	Campbell 2014 log_2_ fold change	Up- or down-regulation
PGSC0003DMT400035006	Chlorophyll a-b binding protein 3 C, chloroplastic	−8.89	−3.17	Same
PGSC0003DMT400034892	Chlorophyll a-b binding protein 3 C, chloroplastic	−14.81	−2.62	Same
PGSC0003DMT400041944	Heat stress transcription factor A-6b	−6.80	−1.59	Same
PGSC0003DMT400026265	Cysteine protease inhibitor 1	−5.65	−2.31	Same
PGSC0003DMT400050249	Chitin-binding lectin 1	−5.95	−1.44	Same
PGSC0003DMT400030387	Chloroplast small heat shock protein class I	−5.41	−2.23	Same
PGSC0003DMT400036565	Flavonol synthase	−6.80	1.42	Different
PGSC0003DMT400076390	Unknown Function	−5.11	−1.78	Same
PGSC0003DMT400034893	Chlorophyll a-b binding protein 3 C	−6.74	−3.27	Same
PGSC0003DMT400062314	Gibberellin 20-oxidase-1	−4.90	3.69	Different
PGSC0003DMT400036925	Unknown Function	−5.06	−4.56	Same
PGSC0003DMT400065197	Nitrate transporter	4.96	1.48	Same
PGSC0003DMT400021584	Beta-1,3-glucanase, acidic	5.47	1.66	Same
PGSC0003DMT400072413	ATPP2-A13	−4.76	−1.16	Same
PGSC0003DMT400078008	17.6 kDa class I heat shock protein	−5.09	−2.72	Same
PGSC0003DMT400082752	Heat stress transcription factor HSFA9	−4.56	−1.82	Same
PGSC0003DMT400061603	ATPase inhibitor	−5.23	−1.91	Same
PGSC0003DMT400078006	17.6 kD class I small heat shock protein	−4.46	−2.41	Same
PGSC0003DMT400063324	Cytochrome P450	−4.43	−1.16	Same
PGSC0003DMT400021234	Heat shock factor protein HSF30	−4.39	−1.50	Same
PGSC0003DMT400060553	GRAS1	4.44	−1.88	Different
PGSC0003DMT400012011	Trichohyalin	13.59	−3.12	Different
PGSC0003DMT400053402	Heat-shock protein	−4.29	−2.04	Same
PGSC0003DMT400043513	Unknown Function	−4.29	5.51	Different
PGSC0003DMT400095387	Unknown Function	−4.30	−1.73	Same
PGSC0003DMT400013627	Auxin-responsive protein IAA16	13.54	4.47	Same
PGSC0003DMT400036981	Acyl carrier protein	−6.16	2.43	Different
PGSC0003DMT400030381	Chloroplast small heat shock protein class I	−4.15	−1.93	Same
PGSC0003DMT400023517	Protein phosphatase 2 C 8	−5.26	−1.56	Same
PGSC0003DMT400073355	SNF4	−4.50	−1.92	Same

Note: These 30 genes were most differentially expressed DEGs between control and heat-stressed potato tubers (sorted from smallest to largest of P-values in our present study) among the DEGs common between our present study on heat-stressed (HS) tubers and the Campbell et al. 2014^16^ study on postharvest non-HS tubers. 24 of 30 genes had the same expression patters of up- or down-regulation between these two studies, significantly different from random events (*P* < 0.05, Chi-Square Test)

Of the heat stress-induced tuber transcriptome of the cultivar Russet Burbank that we observed, 360 DEGs were the same as reported between sprouting and dormant tubers of the cultivar Favorita^17^ (Table S5). The fold changes of DEGs between HS/CK ‘Russet Burbank’ tubers that we observed and sprouting/dormant tubers of Favorita^17^ were also significantly correlated (*R* = 0.23; *P* = 2.93E-14) (Table S4).

There were 88 DEGs shared between our study and previous Campbell et al.^16^ and Li et al.^17^ studies for non-heat-stressed postharvest sprouting versus dormant tuber transcriptomes of ‘Russet Burbank’ and ‘Favorita’ (Table S6). A gibberellin 20-oxidase-1 gene (PGSC0003DMT400062314) and an auxin-responsive protein IAA16 gene (PGSC0003DMT400013627) were among the most differentially expressed genes in terms of log_2_ ratios (Table 1 and Table S6). However, the direction (up or down) of regulation was not the same for all shared DEGs among these three studies.

### KEGG pathway enrichment analysis for differentially expressed genes

**Fig. 4:**
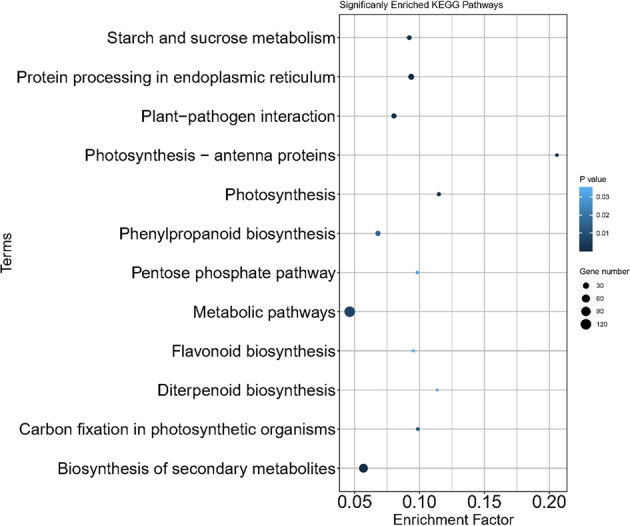
Twelve biological pathways enriched in heat-stressed-tuber transcriptome of ‘Russet Burbank’. Analysis: KEGG pathway enrichment analysis. Genes: 1201 differentially expressed genes (DEGs) between heat-stressed tuber transcriptome and the control tuber transcriptome. DEGs sequences were extracted from Solanum_tuberosum.SolTub_3.0.cdna.all.fa (http://solanaceae.plantbiology.msu.edu/) using TBtools^46^ and analyzed using KOBAS^45^. Plotted with ggplot2 package in R-program. All these 12 pathways were significantly enriched (*P* < 0.05) according to hypergeometric distribution analysis by KOBAS^45^. The dot size represented the number of the DEGs belonging to each pathway. Enrichment Factor means the ratio between the number of DEGs and all genes which were grouped into each pathway. The DEG number and *P* values are presented in Table S7

The 1201 genes differentially expressed between HS and CK ‘Russet Burbank’ tubers were involved in 92 KEGG pathways (data not shown). The following 12 pathways were enriched in HS treatment tubers (Fig. 4): Protein processing in endoplasmic reticulum, Biosynthesis of secondary metabolites, Photosynthesis—antenna proteins, Photosynthesis, Starch and sucrose metabolism, Plant-pathogen interaction, Metabolic pathways, Carbon fixation in photosynthetic organisms, Phenylpropanoid biosynthesis, Diterpenoid biosynthesis, Pentose phosphate pathway, and Flavonoid biosynthesis (Table S7).

**Fig. 5:**
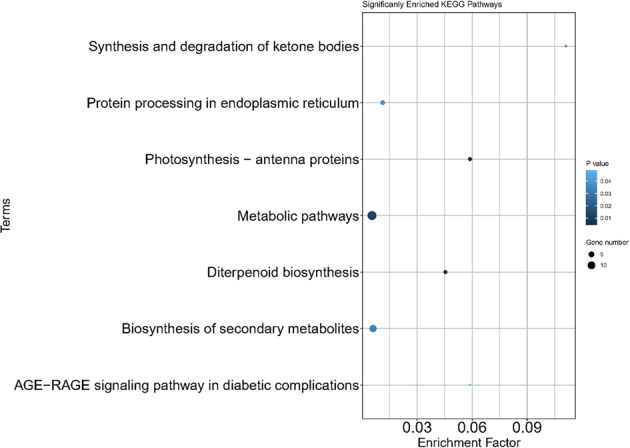
Pathways enrichment of the 88 differentially expressed genes (DEGs) shared by our present study and two previous studies^16,17^ for normal dormant versus postharvest sprouting potato. The dot size represented the number of the DEGs belonging to each pathway. Enrichment factor means the ratio between the number of DEGs and all genes which were grouped into each pathway. KEGG pathway enrichment used KOBAS^45^ ( |log_2_Foldchange | > = 1; *P* < 0.05). The DEG number and *P* values are presented in Table S8

The KEGG pathway enrichment analysis of the 88 DEGs revealed that the following seven pathways were significantly enriched (*P* < 0.05): Photosynthesis—antenna proteins, Diterpenoid biosynthesis, Metabolic pathways, Synthesis and degradation of ketone bodies, Biosynthesis of secondary metabolites, Protein processing in endoplasmic reticulum, and AGE-RAGE signaling pathway in diabetic complications (Fig. 5; Table S8).

The enriched KEGG pathway “Diterpenoid biosynthesis” has two genes involved in gibberellin synthesis/metabolism: PGSC0003DMT400004597 (Ent-kaurenoic acid oxidase) and PGSC0003DMT400062314 (Gibberellin 20-oxidase-1) (Fig. 5). Both genes are important for gibberellin synthesis and plant development^22^. For example, it is known that gibberellin 20-oxidase plays an important role in the GA catabolic pathway and regulate plant stature in plants^23^. PGSC0003DMT400004597, ent-kaurenoic acid oxidase, gene was upregulated in both the previous studies on postharvest sprouting^16,17^ and our study on heat stress response. Obviously, the gibberellin synthesis/metabolism is very active in both heat-stressed tubers and postharvest sprouting tubers.

### GO enrichment analysis of differentially expressed genes

**Fig. 6:**
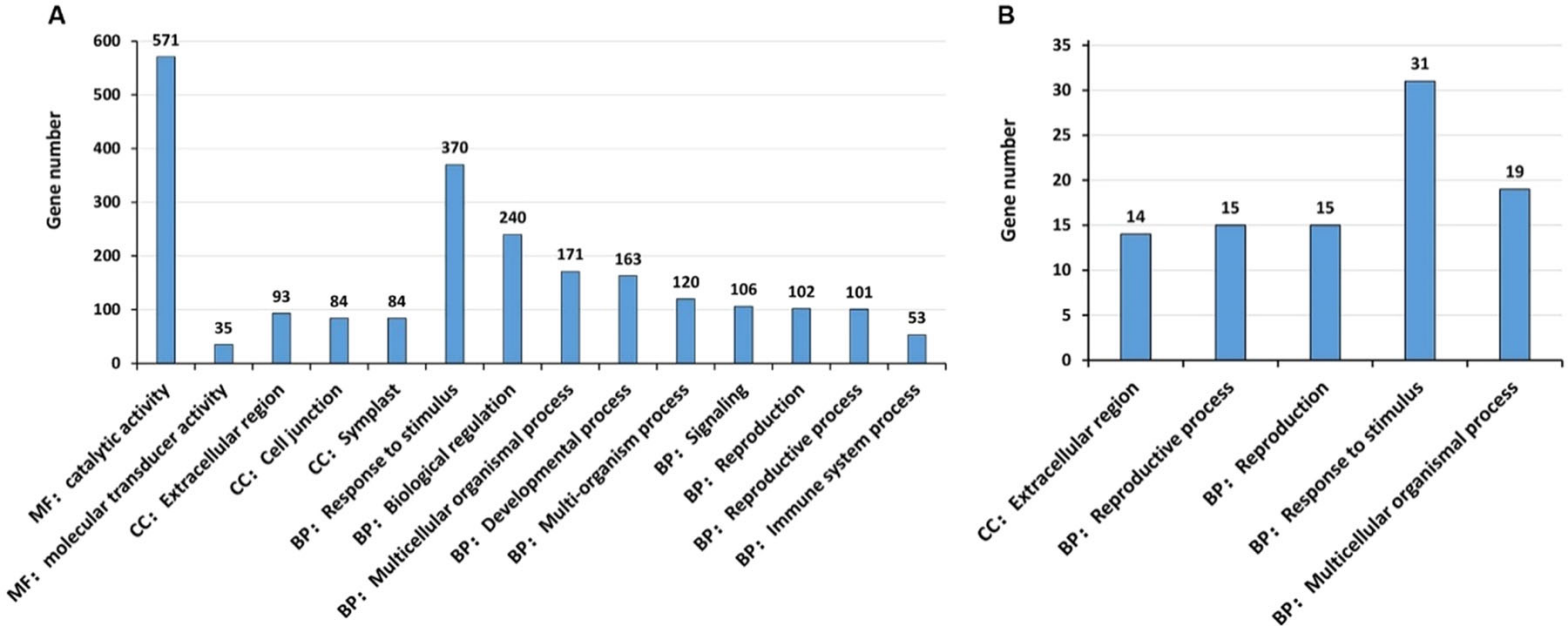
Gene ontology (GO) enrichment analysis of differentially expressed genes (DEGs). **A** 1201 DEGs from our present study on heat stressed tubers of ‘Russet Burbank’. The DEG numbers and *P* values are presented in Table S9. **B** 88 DEGs shared among Campbell et al.^16^ and Li et al.^17^ and this study. The DEG numbers and *P* values (cut by *P* < 0.01) are presented in Table S10. *CC* Cellular Component, *BP* Biological Process, and *MF* Molecular Function

From the 1201 DEGs, the GO term enrichment analysis revealed that 394 GO terms were enriched (*P* < 0.01), and the following 14 terms were level-2 GO terms (*P* < 0.01): MF: Catalytic activity; MF: Molecular transducer activity; CC: Extracellular region; CC: Cell junction; CC: Symplast; BP: Response to stimulus; BP: Biological regulation; BP: Multicellular organismal process; BP: Developmental process; BP: Multi-organism process; BP: Signaling; BP: Reproduction; BP: Reproductive process; and BP: Immune system process (Fig. 6A; Table S9). The terms “Extracellular region” and “Response to stimulus”, and “Signaling” are expected to have enrichment because the heat stress treatment. “Cell junction” and “Developmental process” and “Reproduction” may also be related to heat stress. Interestingly, the GO term “Immune system process”, including various disease resistance-related proteins was also enriched. In the term “Response to stimulus”, 27 DEGs were heat shock proteins.

GO (gene ontology) enrichment analysis of the 88 DEGs shared among our study and two previous sprouting tuber transcriptomes^16,17^ revealed that five level-2 GO terms were significantly enriched (Fig. 6B; Table S10). These five significantly enriched terms are as follows: Extracellular region; Reproductive process, Reproduction, Response to stimulus, and Multicellular organismal process. A Gibberellin 20-oxidase-1 “PGSC0003DMT400062314” was found in all these enriched GO terms except the GO term “Extracellular region”. The GO terms “Response to stimulus” and “Extracellular region” were enriched likely because the heat stress. The enriched GO terms about reproduction was likely related to cell division.

### Heat response element analysis of promoter regions

In the 2,000 bp region upstream of DEGs, 79 of the 88 DEGs carried one or more heat inducible elements of ABRE, G-box, GC-motif, and HSE (Table S11). On average, each of these 79 DEGs had 3.35 ABRE element, 2.56 G-boxes, 0.09 GC-motif, and 0.82 HSE (Table S11). This richness in heat response elements may partly explain why these genes were differentially expressed under heat stress in our present study. Since initiation of potato sprouting does not need heat stress, the differential expression of these genes during postharvest sprouting is expected to be driven by factors other than heat stress. We observed that of these 79 DEGs, 46 were upregulated and 33 were downregulated, even though they all have one or more heat responsive elements (Table S11).

### Protein interaction network

**Fig. 7:**
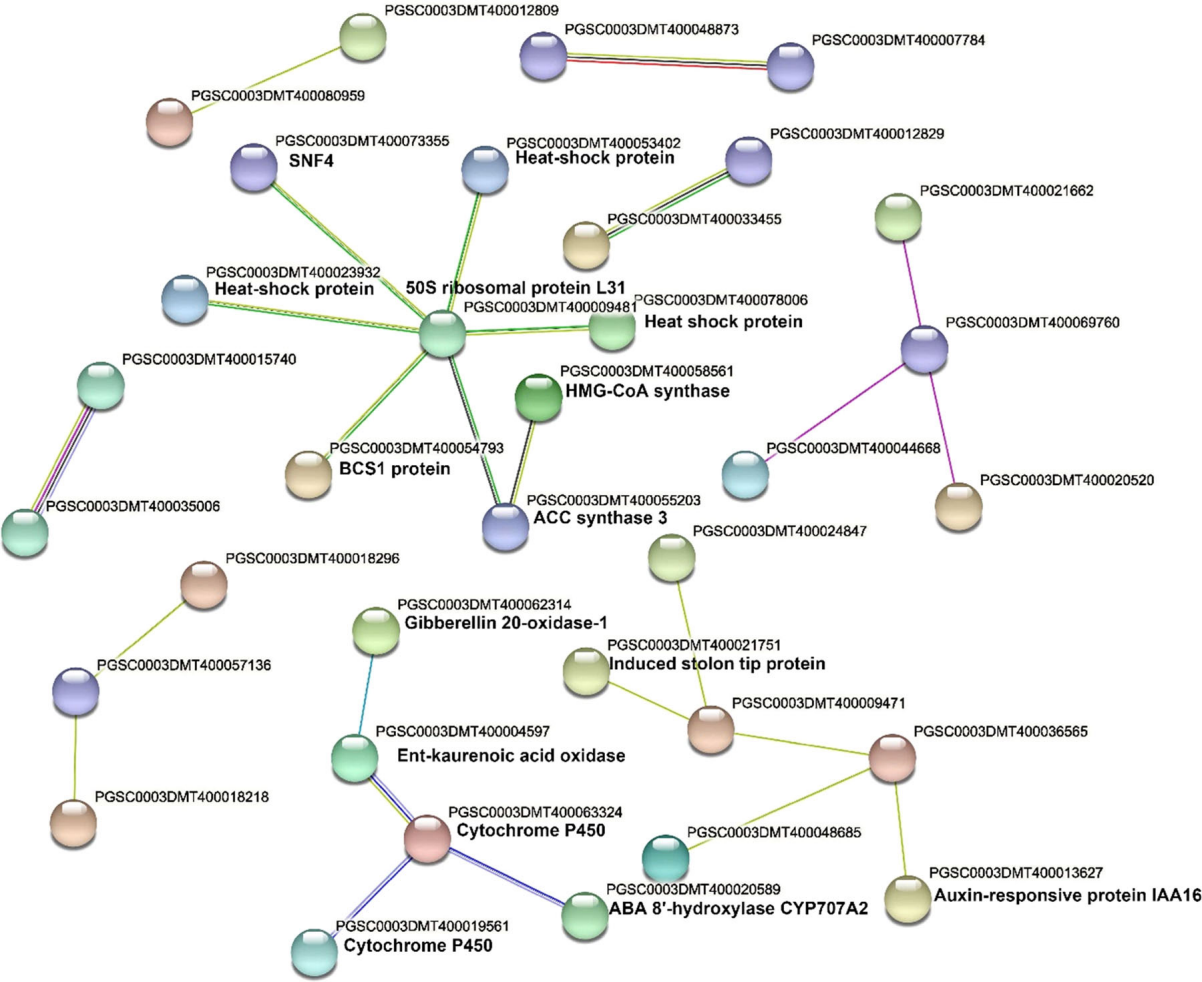
Clusters from functional network analysis of the 88 differentially expressed genes shared between our study on heat stressed tubers and postharvest sprouting tubers. The 88 DEGs were shared among Campbell et al.^16^ and Li et al.^17^ and this study. The network clusters were identified by STRING software (https://string-db.org/cgi/network.pl)

Functional network (Fig. 7) of the proteins encoded by the 88 DEGs that were shared among our study on HS tuber transcriptome, Campbell et al.^16^ and Li et al.^17^ studies on dormant versus postharvest sprouting tubers. Several interaction clusters are very likely involved in sprouting. For example, the cluster on the right bottom area of Fig. 7 includes PGSC0003DMT400013627, an auxin-responsive protein IAA16 and PGSC0003DMT400021751, which is an induced stolon tip protein. Functional network analysis found several clusters that are likely involved in dormancy and sprouting, such as the clusters having auxin-responsive proteins IAA16 and ethylene synthesis-related ACC synthase, and gibberellin oxidase genes (Fig. 7). A 50 S ribosome protein L31 gene plays likely an important role in interacting with heat shock proteins (Fig. 7) and mitochondrial energy production-related gene SNF4.

A cluster includes PGSC0003DMT400020589, which encodes a cytochrome P450, ABA 8′-hydroxylase CYP707A2, gibberellin 20-oxidase, and ent-kaurenoic acid oxidase (Fig. 7). It is known that ent-kaurenoic acid oxidase, which is also a member of the P-450 monooxygenase family, catalyzes a key step in gibberellins (GAs) biosynthesis (https://www.uniprot.org/uniprot/Q9C5Y2). The gene PGSC0003DMT400004597 encoding an ent-kaurenoic acid oxidase was up-regulated in all the three studies: our study on heat stressed tubers and the Campbell et al.^16^ and Li et al.^17^ on postharvest sprouting (Table S6). This cluster has also CYP707A2, an ABA 8′-hydroxylase, which is indispensable for proper control of seed dormancy and germination in Arabidopsis^24^. This gene was also upregulated in all these three studies.

Interestingly, although 79 of the 88 DEGs were enriched in heat inducible elements, carrying 6.82 elements on average (Table S11), ABA8′-hydroxylase (PGSC0003DMT400020589) and ent-kaurenoic acid oxidase (PGSC0003DMT400004597) do not carry any heat-responsive motif (Table S11). The upregulation of these two genes under heat stress may be driven by other functionally connected proteins. The cytochrome P450 gene (PGSC0003DMT400063324) that interacts with these two genes in the functional network carries ABRE, G-box, and GC-motif heat inducible motifs (Table S11). These three genes–ABA8’-hydroxylase, and ent-kaurenoic acid oxidase, and cytochrome P450–are likely key candidate genes involved in dormancy release and tuber sprouting (both heat sprouting and postharvest sprouting).

## Discussion

### Effects of heat stress alone on inducing sprouting in growing tubers

We unequivocally demonstrated that heat stress alone could produce sprouts in growing potato tubers. Previous heat sprout studies were mainly reported from comparisons between tubers from spring-grown potatoes (as control) and summer-grown potatoes (as heat stress treatment)^4,25^. In these field studies, the effect of spring versus summer temperatures on sprouting was most likely confounded with other conditions, such as moisture stress, photoperiod, atmospheric humidity. Also in one previous study on tuber sprouting, potato plants were grown in quartz-filled pots in a greenhouse^14^. Because sand does not hold water well, the authors reported that plants had some wilt during the high temperature period^14^. To our knowledge, our study is the first one where the effect of heat stress alone was evaluated on potato tuber sprouting. We tested tubers from plants growing under sensor-controlled air humidity and in specially designed potting mix to ensure that there was no drought stress. We did not observe any heat sprouts in control plants of any of the 18 cultivars, grown without heat stress treatment, whereas seven of the 18 cultivars grown under high temperatures had heat sprouts. The differences were statistically significant (*P* < 0.05). These results (Table 1 and Fig. 2) demonstrated that heat stress alone without water deficiency can cause heat sprouts in potato.

### Variation in heat sprouting among cultivars

Our study suggests that there was variation for heat sprouts production among the 18 potato cultivars studied. Eleven cultivars did not produce heat sprouts. Of particular interest is cultivar Innovator, one of the most important cultivars for potato production in Canada, which did not produce any heat sprouts. ‘Innovator’ is also relatively tolerant to heat stress for tuber mass^1^. These findings have significance for selection and breeding of potato cultivars that could be grown under global warming conditions without much adverse effect on production. Since limited number of tubers were produced from the eight plants under heat stress treatment, more research is required to verify whether Innovator is also tolerant to hot summer in the field.

Van Ittersum et al.^14^ reported that short dormancy was observed in tubers of the cultivar Diamant but not in a medium-late cultivar Désirée^14^. We demonstrated that heat stress significantly shortens the dormancy period, but with great variation in dormancy period of the heat stressed tubers among the 18 cultivars (Fig. 2). The results of our study in conjunction with a previous study^14^ indicate that genetic variation exists among cultivars for heat-stress sprouting and suggest that genetic improvement for heat stress tolerance in potato can be possible.

### Cultivar maturity and heat sprouting

Whether early maturity or late maturity cultivars have a higher tendency for heat sprouting is one of the questions that does not have a clear answer in the literature, likely because previous studies usually used one^6^, two^25^, or a few cultivars^12^. Heat sprouting in field crop was reported to be more frequent in late-maturing potato plants (seedlings) when naturally-pollinated seeds of the Katahdin and inbred Triumph parents were used and approximately three tubers per plant (genotype) were tested in a field of Louisiana, USA, 1941^4^. Our results using the 18 cultivars do not support this finding because we observed heat sprouts in early, intermediate and late maturing cultivars without significant maturity-period-related pattern. We believe that the number of tubers (15 to 50) produced by heat stressed plants per cultivar in our present study was not very small, and this number should allow a reasonable estimation of the relative frequency of heat sprouting. Furthermore, the tuber number when pooled for each maturity group was much greater than for individual 18 cultivars. We started the heat treatment of all cultivars equally before tuberization but the study of LeClerg and Henderson^4^, was conducted under natural weather condition, and early cultivars likely had tubers already when the hot day time commenced.

Even though we cannot completely rule out the possibility whether maturity and heat sprout tendency have certain correlation or not, at least we can conclude that late cultivars do not have higher tendency to produce heat sprouts and that the correlation between heat sprout tendency and cultivar maturity grouping is quite weak if there is any.

In agriculture, whether heat stress is at the early stage or the late stage of plant development depends on whether potato crop is planted in spring or the fall. In the spring crop, weather becomes hotter gradually during the plant growth. In the fall crop, day temperature gradually become cooler during the plant growth. Our study was conducted under controlled conditions remaining the same during the entire period of tuberization and growth. As such, the response of potato cultivars in the field may be somewhat different because greenhouse and field conditions are not usually identical, but the underlying genetic mechanisms, biological processes, and metabolic pathways for heat-induced sprouting should be the essentially the same between theses two conditions. Therefore, our results should have high significance for understanding the biology of heat sprouting and breeding cultivars tolerant to heat sprouting.

### Effects of heat stress on postharvest dormancy

How to store or use heat-sprouted tubers will partly depend on whether the heat-sprouts continue to grow after harvest, but little information is available about whether heat sprouts continue to grow after the tubers are harvested, and whether heat stress affects the postharvest dormancy period. Our results suggest that heat sprouted tubers become dormant like non-heat stressed tubers after harvest and that heat stress shortens the postharvest dormancy period. Therefore, heat-sprouted tubers can still be stored after harvest for a certain period, but farmers and processors should know that these tubers have shorter dormancy and should be utilized earlier than postharvest unstressed potatoes. In our study, all tubers from heat stressed plants became dormant after harvest and re-sprout after about two months of storage. It is unclear whether becoming dormant was due to removing tubers from plants or due to stress of the exposure to low air humidity after harvest, but the tubers restart to sprout after certain period of storage under the same humidity and temperature conditions after harvest. Our results are consistent with previous reports that heat sprouted tubers become dormant after harvest^11^. However, we could not determine whether heat sprouts could grow into new shoots if the tubers were left in the same pots because we did not test it. Our results could not rule out the possibility that some heat sprouts may continue to grow after tuber harvest if the heat sprouts were sufficiently large. Because heat-sprouted tubers became dormant after harvest in both our study and the previous study^12^, heat sprouting and storage sprouting may involve somewhat different biological mechanisms.

Our results reveal that cultivar ‘Innovator’ stands out for long postharvest dormancy because it did not have any sign of sprouting after 63 days of storage, and only about 50% of the tubers sprouted on the 106^th^ day of storage. Since this cultivar did not have any heat sprouts, is less sensitive to heat-stress induced reduction in productivity and shortening of dormancy, this cultivar should be more suitable than the other 13 cultivars (Fig. 2) for potato production without heat sprouts under climate warming conditions.

### Genic responses to heat stress and comparative transcriptomics for gene regulation correspondence between heat stressed and non-heat-stressed postharvest sprouting tubers

We have established that heat stress can cause sprouting of growing tubers. And non-stressed postharvest tubers usually sprout after a period of dormancy. Therefore, we expect that there should be some correspondence for regulation of certain genes and biological processes and enrichment of pathways between heat-stressed tubers and non-stressed post-dormancy sprouting tubers. Our study addressed this question for the first time and indeed the results from both quantitative RT-PCR of candidate genes and RNA-seq of whole transcriptome in comparison with the previously reported differentially expressed genes between postharvest sprouting and postharvest dormant tubers^16,17^ support our expectation/hypothesis. In addition, we have identified genes, pathways, and biological processes responsive to heat stress in potato.

The gene expression pattern of dormancy marker genes in HS treatment tubers that we observed was similar to that reported by Liu et al. (2017)^15^ for dormancy-released postharvest non-HS stored potato tubers. Liu et al.^15^ reported downregulation expression of the genes *DOG1* and *SLP* and upregulation of *CYP707A1* gene in sprouted postharvest tubers in comparison with dormant postharvest tubers. Interestingly, we also observed that *DOG1* and *SLP* genes had higher expression in control potatoes (non-stressed tubers) in all four cultivars tested (‘Atlantic’, ‘Nipigon’, ‘Russet Burbank’, and ‘Shepody’) and lower expression in heat-stressed tubers of these same four cultivars (Fig. 3). Second-generation RNA sequencing also detected downregulation of these two genes in the transcriptome of the heat stress treatment tuber of ‘Russet Burbank’ (Fig. 3). The *CYP707A1* gene had higher expression in heat-stressed tubers of two (‘Atlantic’ and ‘Russet Burbank’) of these four cultivars tested (Fig. 3). These results suggest that heat stress alone without water-deficiency stress reduced the activities of certain dormancy-related genes and activated sprouting-related genes, which likely enabled the heat-stressed tubers more ready to sprout than the control tubers (Fig. 2). Taken together, the previous postharvest sprouting study^15^ and our present heat-stress study, the results suggest that the *DOG1* and *SLP* genes are involved in heat stress response, dormancy and post-dormancy sprouting, and can be used as marker genes for monitoring dormancy status in both postharvest tubers and heat stress grown tubers. Downregulation of these two genes in potato tubers is likely a sign for potato tubers’ tendency to sprout.

The whole transcriptome analysis of differentially expressed genes (DEGs) between tubers of non-heat-stressed control and tubers of heat-stressed plants of cultivar Russet Burbank in this study and comparative transcriptomic analysis with DEGs between unstressed postharvest dormant and post-dormant sprouting tubers of cultivars Russet Burbank^16^ and Favorita^17^ reported in previous studies, suggest high similarities in regulation of certain genes between heat-stressed tubers and unstressed postharvest sprouting tubers. This similarity in gene expression is strongly supported by highly significant correlations between the fold change in the expression levels of 190 DEGs common between Campbell et al.^16^ and our study and of 360 DEGs common between Li et al.^17^ and our study, and the same direction (up- or down- regulation) of 24 of the 30 top most differentially expressed genes common between our and Campbell et al.^16^ studies for the same cultivar Russet Burbank (significantly different from random events, *P* < 0.05, Chi-Square test, see Table 1). Furthermore, the up or down-regulation direction of the fold change for 56 of the 88 DEGs common among our and Campbell et al.^16^ and Li et al.^17^ studies was the same (Table S6). Furthermore, the correlation of the Log_2_ fold changes of these 88 differentially expressed genes was highly significant between our study and those of Campbell et al.^16^ and Li et al.^17^ studies. Such a high significant agreement for DEGs between HS/CK tuber transcriptome (our study) and postharvest sprouting/dormant tuber transcriptome^16^ cannot be by chance alone.

We recognize that we did not have biological replicates for our whole transcriptome sequencing and DEG analysis. However, this does not affect our conclusions; first because the RNA-Seq results for selected 9 genes were verified by RT-ddPCR analysis, and second and most important high similarities in DEGs between our and previous studies^16,17^ which had biological replicates. Not only DEGs were found to be significantly correlated at the fold change level but also much more impressively for the direction whether a differentially regulated gene was upregulated or downregulated. It must be noted that we extracted RNA from the tuber, which is full of starch, and it is not easy to extract high quality and quantity RNA from tissues rich in starch. On the other hand, RNA preparation in Campbell et al.^16^ and Li et al.^17^ studies were made from the sprout meristems/eyes/buds.

GO terms and KEGG pathways enriched in HS/CK plant tubers in our study for 88 DEGs common between our and two previous studies are consistent with the physiological roles of DEGs and differentially regulated pathways and GO terms. Here we discuss a few selected enriched pathways and GO terms.

The KEGG pathway enrichment analysis of the 88 DEGs common between this study on heat stress and previous studies on postharvest sprouting^16,17^ detected at least two gibberellin synthesis-related genes (encoding ent-kaurenoic acid oxidase, gibberellin 20-oxidase-1) in the enriched diterpenoid biosynthesis pathway (Fig. 5; Table S8), suggesting that gibberellin synthesis/metabolisms were very active in both heat-stressed tubers and postharvest sprouting tubers, likely a common feature related to dormancy release and both heat sprouting and postharvest sprouting. The KEGG pathway enrichment analysis of the 88 DEGs also revealed that not only protein processing and signaling pathways and the hormone-related diterpenoid biosynthesis pathway were enriched but the photosynthesis—antenna proteins pathway was also enriched (Fig. 5; Table S8). Since this enrichment is common in all these three studies, it suggests that the “photosynthesis—antenna proteins pathway” is involved in HS/CK and dormancy/sprouting of tubers.

Potato tuber is a modified stem, and tuber sprouting is essentially the formation and growth of buds; therefore, is expected to be under regulation of plant hormones such as gibberellin and auxin^26^. The auxin-responsive protein IAA16 gene (PGSC0003DMT400013627) was among the DEGs and was upregulated in both our present study (log_2_ ratio was 13.54) and the previous study (log_2_ ratio was 4.47)^16^ in the same cultivar Russet Burbank (Table 1). This gene was downregulated in the previous study on ‘Favorita’ tuber sprout transcriptome^17^. The regulation of this is likely different in different cultivars and in tubers under different treatments. The plant hormone genes or hormone-responsive genes were detected in DEGs common to this heat stress study and the two previous studies on sprouting, which suggest that plant growth regulator pathways likely play an important role in both heat sprouting and postharvest sprouting of potato tubers.

The heat shock transcription factors and heat shock proteins have multiple functions in plant development and growth^27,28^. Interestingly, one heat shock transcription factor and five heat shock proteins are shared DEGs between our study on HS/CK tuber transcriptomes and the previous study on dormant/postharvest sprouting of the same cultivar (Russet Burbank) among the 30 most differentially expressed genes (sorted by *P* values) (Table 1). All these six genes were downregulated in both studies (Table 1), suggesting that these genes must be downregulated in tubers growing under heat stress and in postharvest normal sprouting tubers in order for these tubers to release dormancy. It is known that there are many heat shock proteins that respond to heat at different times in potato^19,29^. Transient silencing of heat shock proteins showed remarkable roles for heat shock proteins during adaptation to stress in plants^30^. Since temperature plays an important role in regulating tuber dormancy and sprouting, it is not surprising that heat shock transcriptional factors and heat shock proteins are involved in these processes. Differential regulation of heat shock protein genes in response to heat stress is a common phenomenon in plants.

The physiological processes common between postharvest sprouting tubers and heat stressed tubers is their tendency to sprout. Postharvest post-dormancy sprouting of tubers is a common phenomenon. Heat stressed tubers had either heat sprouts or much shorter dormancy than the control tubers as shown in our study, suggesting that heat stress might have induced certain mechanism at the gene expression level in heat stressed tubers for them to sprout.

Among the 1201 DEGs that we identified in response to heat stress, 1011 were not shared with DEGs identified previously in response to postharvest sprouting tuber^16^ for the same cultivar Russet Burbank. It is expected that growing tubers during heat stress and postharvest tubers during storage have many differences including tuber aging, water loss, respiration-related sugar metabolism, or simply tissue differences between bud areas and non-bud areas. It is not surprising that most of the 1201 DEGs from our present study were not directly related to dormancy and sprouting and not shared with the postharvest sprouting transcriptome^16^. Heat-stressed immature tubers generally do not have these issues but have other stress response factors. GO analysis of these 1201 DEGs identified that the most enriched group is “Extracellular region”, which consisted of 93 enrichment-detected DEGs (Fig, S1; Table S9). It is not surprising because component of membranes may be among the first cellular components sensing the heat stress signals. It is known from our previous study that in the protein to protein interaction network in human cells, the most important proteins for sensing signals and forwarding signals are mainly in the cell membranes^31^. It is known that increased sucrose synthase activity and decrease invertase activity favour starch and dry matter accumulation in sweet potato and potato^32,33^. Starch and sucrose metabolism was among the enriched KEGG pathways (Fig. 4, Table S7), the upregulation of invertases, cellulases, and amylases and decrease of sucrose synthase were likely responsible for heat-stress reduced decrease of tuber yield but contributed to generate cellular energies to adapted to the stress conditions.

### Heat response and functional interaction network

Heat shock transcriptional factors are known to respond to heat stress very rapidly, for example, some of them reached the peak activity at two hours of heat stress treatment^34^. We found that DEGs carrying heat-responsive elements can be either upregulated or downregulated, depending on the genes, under continued heat stress treatments (Table S11). Because the activity of a gene varies over time according to the function of the gene in plant response to heat stress, it is not surprising that DEGs in the functional network may be upregulated or downregulated at different times.

The ABA8’-hydroxylase (PGSC0003DMT400020589) and ent-kaurenoic acid oxidase (PGSC0003DMT400004597) do not carry any heat-responsive motif (Table S11). The upregulation of these two genes under heat stress may be driven by other functionally connected proteins. The cytochrome P450 gene (PGSC0003DMT400063324) that interacts with these two genes in the functional network carries ABRE, G-box, and GC-motif heat inducible motifs (Table S11). These elements are known to be heat responsive^35^.

The ABA8’-hydroxylase and ent-kaurenoic acid oxidase are hormone pathway genes and do not have any known heat responsive elements but were upregulated in both heat stressed (this study) and postharvest sprouting tubers^16,17^, suggesting that these two genes are likely key candidate genes involved in dormancy release and tuber sprouting (both heat sprouting and postharvest sprouting).

The significant correlation in log_2_ fold changes of shared DEGs between the previous sprouting tuber transcriptome^16^ and the present heat stress tuber transcriptome of the cultivar Russet Burbank can be an effective tool to identify genes that are very likely related to dormancy/sprouting (Table 1). Since heat sprout or heat-induced shortening of dormancy are not processes identical to postharvest sprouting processes, some genes shared between heat stressed tubers and postharvest sprouting tubers but with opposite direction of their regulation (up versus downregulations) can be very interesting because these genes might be candidates for investigating mechanisms involved differentially between the heat stress induced sprouting and postharvest storage-induced sprouting. Nevertheless, our study contributes significantly to the understanding of gene expression responses of growing potato tubers to heat stress and similarities between heat-induced versus postharvest sprouting-induced gene expression and pathways in potato. Our study has also generated transcriptome resource for the global potato genomics community.

### Carbohydrate metabolism, processing quality, and heat tolerance of ‘Innovator’

The cultivar Innovator originated from a cross between ‘Shepody’ and ‘RZ-84-2580’^36^. Both ‘Shepdoy’ and ‘Innovator’ have relatively high tolerance to heat stress in terms of tuber weight^1^, but ‘Innovator’ tubers can be stored for year-round supply. However, ‘Shepody’ is usually used for early-supply and rarely used for year-round supply of potato. ‘Innovator’ normally starts to sprout after 90 days of cold storage (6 to 8 °C) plus 15 days of reconditioning at 15 °C^37^. In a study of sprouting by storage at 15 °C and 95% humidity, 50% of tubers of ‘Innovator’ started to sprout on the 85^th^ day, and 50% of tubers of ‘Russet Burbank’ on the 89 day^38^. We observed that there was no sprouting in ‘Innovator’ tubers, regardless of whether the tubers were from the control or heat stress treatment, at 63day storage at room temperatures. Thus, our study and the previous two studies^37,38^ together indicated that ‘Innovator’ tubers have relatively long dormancy, which is an important trait for cold storage of tubers for French fry processing or room temperature storage in kitchens.

Compared to ‘Shepody’ tubers, ‘Innovator’ tubers have higher total starch content in dry matters, lower protein content, lower glucose content, lower apparent amylose in starch, lower phosphorus in starch, and higher resistant starch in dry matter, according to one of our previous studies of these cultivars^39^. However, compared to ‘Russet Burbank’ (a heat-sensitive cultivar) for these traits, ‘Innovator’ is significantly different only at higher total starch (74.2% vs. 68.9%) in dry matter, higher phosphorus (23.4% vs. 16.8%) in starch and higher resistant starch in dry matter (47.5% vs. 38.5%)^39^. These differences between the two cultivars suggest that further research is needed to clarify whether higher total starch in dry matter, higher phosphorus in starch and higher resistant starch in dry matter can be involved in the higher heat tolerance of ‘Innovator’ than that of ‘Russet Burbank’.

The main processed potato product is fries, the second most important product is potato chips, the third in importance is potato starch^40^. Storage temperatures of potatoes can greatly influence the reducing sugar content, which is an important processing quality trait for fries and chip potatoes^33^. Both ‘Innovator’ and ‘Russet Burbank’ are French fry cultivars with relatively long dormancy^38^, and have shown to have low sugar content in their stored tubers^39^. The reducing sugar content in ‘Innovator’ tubers is known to be also greatly influenced by storage temperatures; for example, tubers under 8 °C had significantly less sugar than tubers stored under 6 °C, followed by 15 days reconditioning at 15 °C^37^. However, most of the 18 cultivars studied in the present investigation were table potatoes. The few processing quality cultivars including ‘Atlantic, ‘Innovator’, ‘Russet Burbank’, and ‘Shepody’ can all be used for both processing and table potatoes. Therefore, our present study used kitchen conditions for storage. Further research is needed to understand the exact mechanisms of heat tolerance and heat sprouting for storage of tubers under processing industry-used temperatures. We observed that certain invertases (PGSC0003DMT400072606) and amylases (PGSC0003DMT400020591) were up-regulated in heat-stressed-tuber transcriptome of ‘Russet Burbank’. The glucose-deprivation-responsive gene SNF4 was down-regulated in heat-stressed-tuber transcriptome (Table 1), which also suggests that heat stress likely increase glucose content in tubers. It is known that these two enzymes play important roles in the production of reducing sugars and influence the processing quality of potatoes^33,41^, and the net activity of acid invertase is positively correlated with glucose content among cultivars^42^. Further research is required to investigate whether editing of these genes can improve the heat tolerance for carbohydrate-related processing quality and heat sprouting traits of potato.

## Conclusions

Heat stress alone can cause heat sprouts in growing potato tubers, and there is substantial variation among cultivars for producing heat sprouts. Heat stress during plant growth shortens the postharvest dormancy period of tubers. Heat sprouting had no clear correlation with cultivar field maturity time. The cultivar Innovator was found to be the most tolerant to heat stress during plant growth in terms of producing heat sprouts and maintaining postharvest tuber dormancy. Heat stress and postharvest non-heat-stress sprouting of potato tubers have certain shared genetic and metabolic mechanisms, evident from the transcriptomics results. Dormancy-associated genes were downregulated in tubers of heat stressed plants in a way similar to that in tubers with postharvest storage-induced sprouting. The expression patterns (up or down regulation and fold change) of many differentially expressed genes in response to heat stress were similar to the expression patterns of DEGs in response to postharvest sprouting of non-heat-stressed tubers. Gibberellin metabolism appears to play a major role in heat sprouting. The identified DEGs can be a useful source for identifying genes responsible for dormancy or sprouting. The information on heat-sprout variation among potato cultivars has significance for developing new potato cultivars tolerant to heat sprouting. Our finding that heat-sprouted tubers became dormant after harvest, but heat stressed tubers had a shorter postharvest dormancy than non-heat-stressed tubers can assist farmers and processors in decision making about how to store and use their heat-stressed potatoes. These results have significance in facilitating parental selection in breeding of heat tolerant cultivars, editing genes to reduce heat sprouting and heat-induced shortening of dormancy, using molecular markers for selection and monitoring heat-sprout tolerance, and developing potato cultivars and integrated measures suitable for sustainable potato production under global climate change (warming) conditions. Our study advances the field of heat stress biology in plants.

## Materials and methods

### Plant materials

Potato (*Solanum tuberosum* L.) tubers in this dormancy/sprouting study were used from our experiment conducted for evaluating heat stress tolerance of 55 cultivars reported previously^1^. From the 55 cultivars, the following 18 potato cultivars were selected for our present study: ‘AC Belmont’, ‘Atlantic’, ‘Cherry Red’, ‘Chieftain’, ‘Denali’, ‘Epicure’, ‘Eramosa’, ‘Goldrush’, ‘Innovator’, ‘Mainechip’, ‘Mirton Pearl’, ‘Nipigon’, ‘Rariton’, ‘Red Cloud’, ‘Russet Burbank’, ‘Russet Norkotah’, ‘Shepody’, and ‘Superior’. These cultivars were used mainly because they produced sufficient number of tubers under heat stress for conducting the present heat sprout and dormancy/storage analysis. ‘Russet Burbank’ produced only one small tuber under the heat stress conditions^1^ but was still used in our present study because ‘Russet Burbank’ has been the most important cultivar for the potato industry for decades in North America^43^.

Four cultivars—Atlantic, Russet Burbank, Shepody, and Nipigon—were used for gene expression analysis by polymerase chain reactions (PCR). The other 14 cultivars were used for dormancy and storage sprouting testing. This is because heat stress treatment greatly reduced tuberization (number and mass of tubers), and tubers from the eight heat-stressed (HS) plants for each cultivar did not have sufficient potato tubers for conducting both molecular analysis and storage dormancy test. Cultivars Shepody and Russet Burbank were employed for differential gene expression analysis by whole transcriptome sequencing (RNA-Seq). Again, the cultivar Russet Burbank was used as one of the four cultivars for gene expression analysis because this cultivar was among the cultivars most sensitive to heat stress^1^ and is the most important cultivar to the French fry industry.

### Plant growth conditions, heat stress treatment, and heat sprouting

As previously described^1^, sixteen plantlets (with one plantlet per pot) of each cultivar were grown in potting mix under 22 °C/18 °C day/night temperatures, 14 h photoperiod and 70% humidity in greenhouses at the Fredericton Research and Development Centre of Agriculture and Agri-Food Canada in Fredericton, New Brunswick, Canada. After three-weeks of growing under these conditions, eight of the 16 plants per cultivar were moved to a next door greenhouse under the same photoperiod and humidity but under a day/night temperature of 35 ± 1 °C/28 ± 1 °C for heat stress treatment (HS) until harvest as described previouly^1^. The other eight plants were kept in this original greenhouse under original growth conditions (22 °C/18 °C day/night temperatures) to serve as control (CK). The potting-mix was consisted of peat moss–perlite–soil–vermiculite at 2:1:1:1, v-v:v:v., for both CK and HS treatments to ensure good holding of humidity in the potting-mix after watering. The harvest was done on the 102^nd^ day after planting in the potting mix. At the harvesting time, plants of a few early maturity cultivars, such as ‘Shepody’, showed signs of leaf yellowing for plants growing under the CK greenhouse conditions but plants of most other cultivars were still green and growing in either CK or HS greenhouse treatment.

At harvest, a cultivar was classified as a sprouted cultivar if at least one tuber from the eight CK or HS plants had a sprout more than 2 mm. If there were no sprouts at harvest in CK plant tubers but sprouts were present in HS potato tubers, the sprouts were called “heat sprouts”. The length of the longest sprout from the HS tubers (no sprouting was observed in the CK treatment) of each cultivar was recorded as the “the longest heat sprout length”.

### Sprouting and dormancy record during storage

We used six large, healthy, and uniform tubers per cultivar for the sprouting test of the CK tubers and a varied number of HS tubers (Fig. 2) during postharvest storage. All tubers were stored at room temperature (air conditioning for 21 °C) under approximately 90% relative humanity in the dark within paper bags placed inside cardboard boxes. The tuber dormancy status was checked approximately every two weeks by briefly opening the bags to quickly check whether there are some sprouts. We did not take out every tuber to check and measure the sprout length because we tried not to disturb and break the sprouts accidentally, which could have affected out data quality. The length of the longest sprout of each tuber was recorded only on the 63 day of storage and the 106 day of storage. This was because there were clear differences on the 63 day between control non-heat stressed (CK) and heat stressed (HS) tubers in sprouting, which allowed us to make unambiguous conclusions. We chose 106^th^ day for recording data because we found that most tubers had sprouted already by that time, and the differences in tuber sprouting rate between cultivars could be less obvious if every tuber was sprouted during further prolonged storage. A tuber was classified as “sprouted” if the tuber had at least one bud that reached 2 mm or longer. Postharvest sprouting during storage was not the regrowth of original heat sprouts.

### RNA extraction and gene expression analyses

Total RNA was extracted from the fresh materials of longitudinal sections of tubers at the time of harvest using RNeasy Plant kit (https://www.qiagen.com, Qiagen, Hilden, Germany) following the manufacturer’s protocol. One tuber from each of the four plants per cultivar was used for RNA extraction. RNA from each tuber was prepared separately. For ‘Russet Burbank’, RNA for the control was a mixture of four tubers at equal amounts of total RNA, whereas RNA for the HS treatment was only for one tuber because only one tuber was formed in all eight plants grown under the HS treatment. For other three cultivars each, the RNA samples used for gene expression analysis for both the CK and HS treatment tubers were mixtures of RNA extracted from three tubers/plants. The quality and quantity of all RNA samples were assessed using a NanoDrop 1000 Spectrophotometer (Thermo Scientific, Waltham, MA, USA). The mixed RNA sample for each cultivar had equal amounts of RNA from three or four plants.

### Gene expression analysis of dormancy marker genes

We used droplet digital qPCR for quantitative analysis of differential expression of previously identified^17^ two dormancy (*DOG1* and *SLP*) and one sprouting (*CYP707A1*) associated genes in control and heat-stressed sprouting tubers. The cDNA from the RNA was synthesized using the SuperScript (Invitrogen, Thermo Scientific). We used primers for *DOG1*, *SLP* and *CYP707A1* genes to absolutely quantify RNA by RT-ddPCR analysis. Two dormancy-associated genes, *DOG1* and *SLP*, are known to have upregulation in dormant tubers and downregulation in sprouting tubers, whereas the sprouting-associated gene, *CYP707A1*, is known to have downregulation in dormant tubers and upregulation in sprouting tubers. *Elfa4* (an elongation factor) gene was used as the endogenous control gene for RT-ddPCR. The primers for the *Elfa* gene were designed by our present study using a conserved region among four transcript variants of this gene. The specific primers for the marker and control genes above were designed using Primer 5 (Version 5.2.0) and primer-BLAST (http://www.ncbi.nlm.nih.gov/tools/primer-blast/index.cgi?LINK_LOC = BlastHome).

The absolute quantification of the reverse transcribed DNA was performed on a QX200 Droplet Digital PCR (ddPCR) System (Bio-Rad, Hercules, CA, USA). The PCR conditions were 95 °C for 5 min, 40 cycles of 95 °C for 30 s, 55 °C for 30 s, and 72 °C for 45 s, then 4 °C for 5 min. According to our previous gene expression analysis, we found that ddPCR results were much more reproducible than real-time quantitative PCR (data not shown). Therefore, after two RT-ddPCR trials to optimize the signal strength by adjusting the fold of dilution of cDNA samples, the data analysis was based the formal experiment without technical repeats.

### RNA sequencing and transcriptome analysis

The whole transcriptome was sequenced using Illumina PE-100bp. The Illumina RNA sequences were used for transcriptome comparison between the CK tubers (mixture of four tubers) and a HS tuber (without heat sprouting) of ‘Russet Burbank’. We also tried to use the cultivar Shepody for RNA sequencing, but the CK library did not meet quality requirement for sequencing, and therefore ‘Shepody’ was excluded from sequencing analysis. Compared to leaves, it is quite difficult to extract high quantity RNA from potato tubers which are rich in starch and polysaccharides.

The quality of all RNA samples was assessed using a NanoDrop 1000 Spectrophotometer (Thermo Scientific, Waltham, MA, USA). Magnetic beads with Oligo-dT were used to purify poly-(A) mRNA from the total RNA. The mRNA was mixed with fragmentation buffer to obtain short fragments. Libraries were constructed by using the TruSeq RNA Sample Preparation Kit (Illumina, San Diego, CA, USA). An Agilent 2100 BioAnalyzer (Agilent Technologies, Palo Alto, CA, USA) was used to assess the quality of the sample libraries. Finally, the libraries were sequenced using the system Illumina HiSeq4000 PE-100bp platform according to the manufacturer’s sequencing protocols. The sequencing was performed by McGill University and Genome Quebec Innovation Centre (Montreal, Quebec).

### Differential gene expression analysis

The Illumina reads were trimmed off the primers, cleaned according to quality control, and the unique reads in each transcript was mapped and identified by the unimap pipeline of TBSPG^21^. Briefly, the raw Illumina reads were trimmed using Trimmomatic to remove the adapters and remove the reads that were shorter than 50 nucleotides or lower than the Phred-33 threshold, then mapped using BWA, identified and remove multi-mapped reads were defined by SAMtools (with the “-F 4” option) and TBSPG building scripts to ensure that only a read having a single mapped location was called a uniquely mapped read. The reference sequence for each gene in mapping during the transcriptome analysis was from the file of PGSC_DM_v4.03_transcript-update representative.fasta.zip, which is the transcript that produces the longest peptide sequence among all the alternative isoforms of a gene (http://solanaceae.plantbiology.msu.edu/pgsc_download.shtml). This unique-mapping approach greatly increases specificity and facilitates discussion because only those reads each mapping to a unique location were counted^21^.

The control tuber transcriptome had 23,822,496 clean and unique reads, mapped uniquely to 25,076 genes (representative transcripts). The HS transcriptome had 24,628,632 clean and unique reads mapped uniquely to 26,783 genes (DMTs). The differential gene expression analysis was performed using DESeq^44^ using a Linux bioinformatics server computer. Two available transcriptional datasets of the postharvest sprouted non-HS potato tubers^16,17^ were used to perform correlation analysis for DEGs that we identified in the heat-stressed tubers of our present study with the shared DEGs in the postharvest post-dormancy sprouting reported earlier^16,17^.

The differential gene expression results from the Illumina sequencing data were validated for 9 genes by absolute quantitation of RT-ddPCR (Table S1). *Elfa* gene was again used as the internal control. The regression analysis between the folds of changes of the expressed genes from the RNA-Seq data and the folds of changes detected by RT-ddPCR was performed (Table S1 and Fig. S1).

### KEGG pathway enrichment analysis

The pathway enrichment analysis was conducted using all 1201 DEGs in the HS tuber transcriptome and using the 88 DEGs shared among our study and the two previous dormant versus sprouting tuber transcriptomes^16,17^ using KOBAS^45^ (|log_2_Foldchange | >=1, *P* value<0.05). A graph plot was developed using the ggplot2 package in R program. The dot size represented the number of the DEGs belonging to each pathway. The enrichment factor meant the ratio between the number of DEGs and all genes which were grouped into each pathway.

### GO enrichment analysis

GO term analysis for the 1201 genes expressed differentially between the heat stressed and control tubers as well as for 88 DEGs common between our study on HS tubers and previous unstressed dormant versus postharvest sprouting tuber studies of Campbell et al.^16^ and Li et al.^17^ was performed using the hypergeometric distribution package in the software TBtools^46^ and the GO terms of genes from the reference potato genome^47^ (*P* < 0.01). At the GO term level 2 of GO Classes, “CC” means Cellular Component, “BP” means Biological Process, and “MF” means Molecular Function.

### Heat responsive element analysis of promoter regions

The 2000 bp promoter region immediately upstream of each of the 88 DEGs that were shared by two previous postharvest sprouting studies (Campbell et al.^16^ and Li et al.^17^) and this study were extracted from Solanum_tuberosum.SolTub_3.0.dna.toplevel.fa. These sequences were analyzed by PlantCARE (http://bioinformatics.psb.ugent.be/webtools/plantcare/html/)^48^. The heat responsive elements ABRE, G-Box, and GC-motif (Heat shock element) were according to Rerksiri et al^35^. HSE (heat sock elements) were searched using the core HSE motif “nGAAnnTTCn”^49^.

### Functional network analysis

Network clusters were identified by STRING software (https://string-db.org/cgi/network.pl) using the 88 DEGs that were shared among Campbell et al.^16^ and Li et al.^17^ and this study.

## Supplementary information

Table S1

Table S2

Table S3

Table S4

Table S5

Table S6

Table S7

Table S8

Table S9

Table S10

Table S11

Figure S1

## Data Availability

The sequence datasets used in our study have been deposited to NCBI (Illumina, NCBI Submission ID: SUB4247015, BioProject ID:PRJNA578671) and are publicly available.
