# Supplementary material for: Heat-stress-induced sprouting and differential gene expression in growing potato tubers: Comparative transcriptomics with that induced by postharvest sprouting
Source: Hortic Res. 2021 Oct 15;8:226. doi: 10.1038/s41438-021-00680-2 (PMC8519922; doi:10.1038/s41438-021-00680-2)
Supplement: Supplementary file 1 — Table S1 [file 41438_2021_680_MOESM1_ESM.docx]

**Table S1. Primers used for validating RNA-Seq results and reverse transcription digital droplet PCR analysis (RT-ddPCR)**

| **Primer ID** | **Primer name** | **Sequence 5'-3'** | **Template ID (DMT#)** | **Function annotation in database #** |
| --- | --- | --- | --- | --- |
| Pr6067 | Elfa4seqsF | CTGTTAAGGATCTGAAGCGTGGT | A conserved of Elfa4 genes | An elongation factor |
| Pr6068 | Elfa4seqsR | AATGTGGGAAGTGTGGCAGTCG |  |  |
| Pr6073 | sHspcF | ATGTCGCCGATGAGAACAATGAG | PGSC0003DMT400008351 | Small heat shock protein, chloroplastic |
| Pr6074 | sHspcR | TGAATGTCCCAAGGAGTGCGTAT | PGSC0003DMT400008351 | Small heat shock protein, chloroplastic |
| Pr6079 | β-amyF | GAATGGGTCCTTGTGGTGAACT | PGSC0003DMT400052839 | Beta-amylase PCT-BMYI |
| Pr6080 | β-amyR | ATCTGCTGATGCTGCTAGTGAAG | PGSC0003DMT400052839 | Beta-amylase PCT-BMYI |
| Pr6085 | HSF30F | CCAGTGCTGAGAACCTTCAAGATG | PGSC0003DMT400021234 | Heat shock factor protein HSF30 |
| Pr6086 | HSF30R | CAACAGAAGCCGACCTGACATTG | PGSC0003DMT400021234 | Heat shock factor protein HSF30 |
| Pr6089 | UbiF | CGTCAGCGGGAATCAATAAAGGAT | PGSC0003DMT400059650 | Ubiquitin |
| Pr6090 | UbiR | CCTGAATGGCAGCCTTAACATCT | PGSC0003DMT400059650 | Ubiquitin |
| Pr6093 | As-peroF | CAAGACAGAACCACCTCCAGAAG | PGSC0003DMT400004360 | Ascorbate peroxidase |
| Pr6094 | As-peroR | CCAGAGTGTGACCACCAGATAAAG | PGSC0003DMT400004360 | Ascorbate peroxidase |
| Pr6127 | NR_F | GGCAGAGCAAATCCCAGATAGAG | PGSC0003DMT400077648 | Nitrate reductase |
| Pr6128 | NR_R | TCATAGGAGGTGGTCCACAAGC | PGSC0003DMT400077648 | Nitrate reductase |
| Pr6465 | DOG1F* | ATACATGGACACCCGCAGACA | PGSC0003DMT400073133* | Delay of germination 1 |
| Pr6466 | DOG1R* | TGCCACTCATCGCATAAAGGA | PGSC0003DMT400073133* | Delay of germination 1 |
| Pr6467 | CYP707A1F* | GTACAGGTGGTCTATGGTGG | DQ206630*/PGSC0003DMT400047537 | ABA 8'-hydroxylase CYP707A1 |
| Pr6468 | CYP707A1R* | CGATGCTTGTTGTCTTGATGTTGATTG | DQ206630*/PGSC0003DMT400047537 | ABA 8'-hydroxylase CYP707A1 |
| Pr6469 | SLPF* | ACGAACGTAGGCGACGCAAAT | PGSC0003DMT400015864* | Cucumisin |
| Pr6470 | SLPR* | CCATCAACCCAAGAAAGTGACCC | PGSC0003DMT400015864* | Cucumisin |

*#: PGSC:* [*http://solanaceae.plantbiology.msu.edu/pgsc_download.shtml*](http://solanaceae.plantbiology.msu.edu/pgsc_download.shtml)*. *: Primers selected from Liu 2017^15^. For CYP707A1, we used the accession number DQ206630 to find the mRNA sequence of this gene from BCNI, then searched for the DMT number by blast tool in PGSC database.*
