# Supplementary material for: Heat-stress-induced sprouting and differential gene expression in growing potato tubers: Comparative transcriptomics with that induced by postharvest sprouting
Source: Hortic Res. 2021 Oct 15;8:226. doi: 10.1038/s41438-021-00680-2 (PMC8519922; doi:10.1038/s41438-021-00680-2)
Supplement: Supplementary file 4 — Table S4 [file 41438_2021_680_MOESM4_ESM.docx]

**Table S4. Correlation between the values of HS/CK log_2_ fold changes of gene expression levels from the heat-stressed tuber RNA-Seq of our present study and the values of sprouting /dormant log_2_ fold changes of gene expression levels of previous studies on postharvest sprouting tubers.**

| **Comparison** | **Correlation** | ***P* *value*** | **Shared DEGs** |
| --- | --- | --- | --- |
| Present HS/CK vs. Sprouting/Dormant ^16^ | 0.20 | 6.12E-11 | 190 |
| Present HS/CK vs. Sprouting/Dormant ^17^ | 0.23 | 2.93E-14 | 360 |
|  |  |  |  |

*CK: control non-stressed, HS: heat-stressed. DEG: Differentially expressed genes, between the heat-stressed and non-heat-stressed tubers in the present study, and between sprouted and non-sprouted postharvest tubers in the two previous studies*^16, 17^*.*
