# Supplementary material for: Heat-stress-induced sprouting and differential gene expression in growing potato tubers: Comparative transcriptomics with that induced by postharvest sprouting
Source: Hortic Res. 2021 Oct 15;8:226. doi: 10.1038/s41438-021-00680-2 (PMC8519922; doi:10.1038/s41438-021-00680-2)
Supplement: Supplementary file 6 — Table S6 [file 41438_2021_680_MOESM6_ESM.docx]

**Table S6. Eighty-eight differentially expressed genes (Log_2_ fold change (Log_2_FC)) shared between heat-stressed tuber transcriptome of the present study and the postharvest sprouting tuber transcriptomes of cultivars Russet Burbank (Campbell et al. 2014) and Favorita (Li et al. 2017)**

| **Transcript ID** | **log_2_FC in the present study** | **Log_2_FC in Russet Burbank (Campbell et al. 2014)** | **Log_2_FC in Favorita (Li et al. 2017)** | **Annotated function** |
| --- | --- | --- | --- | --- |
| PGSC0003DMT400000089 | -3.23 | -1.54 | -1.12 | Acetyl-coenzyme A carboxylase carboxyl transferase alpha |
| PGSC0003DMT400001233 | -2.95 | -2.31 | 6.79 | Zinc finger protein |
| PGSC0003DMT400002085 | 3.49 | 1.62 | 4.68 | Extensin (ext) |
| PGSC0003DMT400002449 | -3.17 | 1.14 | 2.76 | Reticulon family protein |
| PGSC0003DMT400004597 | 3.87 | 1.81 | 2.34 | Ent-kaurenoic acid oxidase |
| PGSC0003DMT400004734 | -2.48 | 1.29 | 3.78 | Conserved gene of unknown function |
| PGSC0003DMT400004791 | 2.94 | 2.10 | 5.69 | Non-specific lipid-transfer protein 1 |
| PGSC0003DMT400006461 | 3.03 | 1.48 | 2.61 | Zinc finger protein |
| PGSC0003DMT400007784 | 2.75 | 1.58 | 3.40 | Kinesin |
| PGSC0003DMT400009196 | 4.42 | -1.79 | 1.45 | Thaumatin protein |
| PGSC0003DMT400009471 | 2.59 | 4.40 | 7.14 | Phenylcoumaran benzylic ether reductase |
| PGSC0003DMT400009481 | -2.55 | 1.52 | 1.07 | 50S ribosomal protein L31 |
| PGSC0003DMT400011910 | 3.85 | -2.15 | 3.07 | Hydroxyproline-rich glycoprotein (HRGP) |
| PGSC0003DMT400012809 | 3.54 | 1.82 | 6.23 | Endo-beta-1,4-glucanase |
| PGSC0003DMT400012829 | 3.26 | 1.40 | 3.81 | UDP-glucose 4-epimerase |
| PGSC0003DMT400013627 | 13.54 | 4.47 | 3.64 | Auxin-responsive protein IAA16 |
| PGSC0003DMT400015740 | -4.15 | -1.88 | 1.51 | Chlorophyll a-b binding protein 4, chloroplastic |
| PGSC0003DMT400016545 | 2.85 | 1.24 | 7.18 | GDSL-like Lipase/Acylhydrolase family protein |
| PGSC0003DMT400017948 | 5.30 | 1.93 | 6.86 | Zinc finger family protein |
| PGSC0003DMT400018218 | 3.97 | 1.43 | 3.05 | Epidermis-specific secreted glycoprotein EP1 |
| PGSC0003DMT400018296 | 3.33 | 1.69 | 2.23 | Epidermis-specific secreted glycoprotein EP1 |
| PGSC0003DMT400019561 | 3.28 | 2.91 | 1.55 | Cytochrome P450 |
| PGSC0003DMT400019863 | -3.45 | 1.80 | 1.48 | Sulfate/bicarbonate/oxalate exchanger and transporter sat-1 |
| PGSC0003DMT400020122 | 2.52 | 2.99 | 6.26 | Xyloglucan endotransglucosylase-hydrolase XTH9 |
| PGSC0003DMT400020314 | 2.37 | 2.67 | 6.38 | Xyloglucan endotransglucosylase-hydrolase XTH9 |
| PGSC0003DMT400020520 | -3.08 | 2.86 | 4.22 | ATP binding protein |
| PGSC0003DMT400020589 | 2.80 | 1.29 | 1.09 | ABA 8'-hydroxylase CYP707A2 |
| PGSC0003DMT400021584 | 5.47 | 1.66 | 2.29 | Beta-1,3-glucanase, acidic |
| PGSC0003DMT400021662 | 3.09 | 2.52 | 2.81 | ATP binding / kinase/ protein serine / threonine kinase |
| PGSC0003DMT400021751 | -5.31 | 3.32 | 8.87 | Induced stolon tip protein |
| PGSC0003DMT400023932 | -2.85 | -4.30 | 1.45 | Small heat-shock protein homolog protein |
| PGSC0003DMT400023962 | -4.27 | 2.09 | 2.89 | Proteinase inhibitor |
| PGSC0003DMT400024338 | 3.07 | 2.78 | 7.39 | UDP-glucosyltransferase |
| PGSC0003DMT400024847 | 2.41 | 1.36 | 2.72 | Aquaglyceroporin (Tonoplast intrinsic protein (Tipa)) |
| PGSC0003DMT400025955 | 2.54 | 2.02 | 6.02 | Patatin 3 |
| PGSC0003DMT400027065 | 2.82 | 1.73 | 9.11 | CHP-rich zinc finger protein |
| PGSC0003DMT400028172 | 4.44 | 2.15 | 7.68 | Lysine/histidine transporter |
| PGSC0003DMT400028656 | 4.71 | 3.70 | 4.21 | UDP-glucuronosyltransferase |
| PGSC0003DMT400029287 | -3.86 | 2.09 | 4.01 | P21-rho-binding domain-containing protein |
| PGSC0003DMT400032789 | 2.69 | 1.45 | 4.99 | Cationic peroxidase |
| PGSC0003DMT400033248 | 3.50 | 1.26 | 3.13 | ESC |
| PGSC0003DMT400033455 | 4.82 | 1.91 | 2.07 | Cellulose synthase CslG |
| PGSC0003DMT400035006 | -8.89 | -3.17 | 2.04 | Chlorophyll a-b binding protein 3C, chloroplastic |
| PGSC0003DMT400036431 | 3.31 | 2.85 | 6.42 | Abhydrolase domain containing |
| PGSC0003DMT400036565 | -6.80 | 1.42 | 3.23 | Flavonol synthase |
| PGSC0003DMT400038362 | 2.55 | 3.65 | 9.55 | EDGP |
| PGSC0003DMT400041049 | 11.48 | 2.93 | 3.37 | Methylketone synthase II |
| PGSC0003DMT400043513 | -4.29 | 5.51 | 3.37 | Conserved gene of unknown function |
| PGSC0003DMT400044668 | 2.93 | 2.11 | 3.61 | Receptor-like kinase |
| PGSC0003DMT400044708 | -4.75 | 2.75 | 8.04 | Conserved gene of unknown function |
| PGSC0003DMT400048685 | 3.04 | 3.08 | 9.34 | Polyphenol oxidase A, chloroplastic |
| PGSC0003DMT400048873 | 11.70 | 1.94 | 4.35 | Kinesin-3 |
| PGSC0003DMT400049875 | -3.39 | 1.81 | 8.72 | Arabinogalactan peptide 20 |
| PGSC0003DMT400053402 | -4.29 | -2.04 | 1.66 | Heat-shock protein |
| PGSC0003DMT400054793 | 3.76 | 1.64 | 4.22 | BCS1 protein |
| PGSC0003DMT400054947 | 2.53 | 1.92 | 3.87 | U-box protein |
| PGSC0003DMT400055203 | 3.51 | 2.54 | 5.33 | 1-aminocyclopropane-1-carboxylate synthase 3 |
| PGSC0003DMT400055426 | -2.86 | 1.92 | 2.74 | Snakin-1 |
| PGSC0003DMT400057136 | 2.96 | 1.18 | 3.28 | Polygalacturonase inhibitor protein |
| PGSC0003DMT400057397 | -4.43 | -2.71 | -1.97 | Conserved gene of unknown function |
| PGSC0003DMT400057515 | 3.25 | 1.88 | 4.94 | Gene of unknown function |
| PGSC0003DMT400058561 | 3.94 | 1.94 | 2.84 | HMG-CoA synthase |
| PGSC0003DMT400059944 | -3.43 | 1.42 | 2.69 | White-brown-complex ABC transporter family |
| PGSC0003DMT400061076 | -3.65 | 3.25 | 2.55 | Globulin |
| PGSC0003DMT400061974 | 3.28 | 2.18 | 6.13 | Peptide transporter |
| PGSC0003DMT400062314 | -4.90 | 3.69 | 1.66 | Gibberellin 20-oxidase-1 |
| PGSC0003DMT400063308 | -3.13 | 2.20 | 3.26 | Flavonol 4'-sulfotransferase |
| PGSC0003DMT400063324 | -4.43 | -1.16 | -1.39 | Cytochrome P450 |
| PGSC0003DMT400064370 | -3.20 | 2.75 | 4.33 | Conserved gene of unknown function |
| PGSC0003DMT400064535 | 5.31 | 2.51 | 4.31 | Leucine-rich repeat receptor protein kinase EXS |
| PGSC0003DMT400064581 | -2.54 | 2.13 | 1.26 | Conserved gene of unknown function |
| PGSC0003DMT400065197 | 4.96 | 1.48 | 3.44 | Nitrate transporter |
| PGSC0003DMT400068267 | -2.62 | 1.28 | 2.11 | Conserved gene of unknown function |
| PGSC0003DMT400068332 | 4.18 | 3.38 | 4.03 | Transcription factor |
| PGSC0003DMT400069760 | -3.20 | -1.41 | -1.11 | Phosphoinositide-specific phospholipase C |
| PGSC0003DMT400069972 | 2.67 | 3.59 | 6.03 | Glucosyltransferase |
| PGSC0003DMT400071781 | -2.37 | -1.84 | 3.09 | Conserved gene of unknown function |
| PGSC0003DMT400071820 | -3.42 | -1.64 | -1.13 | ER lumen retaining receptor family |
| PGSC0003DMT400073355 | -4.50 | -1.92 | -1.17 | SNF4 |
| PGSC0003DMT400075057 | -4.92 | -2.39 | -1.03 | Ferritin |
| PGSC0003DMT400076784 | -6.16 | 2.59 | 4.26 | Conserved gene of unknown function |
| PGSC0003DMT400078006 | -4.46 | -2.41 | 1.23 | 17.6 kD class I small heat shock protein |
| PGSC0003DMT400078609 | 2.86 | 1.76 | 8.41 | Proteinase inhibitor IIa |
| PGSC0003DMT400079203 | 2.95 | 2.58 | 7.12 | Glycosyltransferase |
| PGSC0003DMT400080959 | 2.50 | 5.43 | 5.43 | Glycine-rich cell wall structural protein |
| PGSC0003DMT400081244 | 2.79 | -1.99 | 8.72 | Phospholipase A1 |
| PGSC0003DMT400081314 | 3.26 | 1.25 | 3.66 | Endo-1,4-beta-glucanase |
| PGSC0003DMT400083791 | 11.70 | -2.20 | 4.63 | Periaxin |

#: Two previous studies^16, 17^. Campbell et al. 2014^16^. Li et al. 2017^17^. PGSC IDs were from <http://solanaceae.plantbiology.msu.edu/>. The correlation of the Log2 fold changes of these 88 differentially expressed genes was highly significant between the present study of heat stressed tubers on ‘Russet Burbank’ and Campbell et al. 2014 on postharvest unstressed sprouting tubers of ‘Russet Burbank’ (*R* = 0.307, *P* < 0.01), highly significant between the present study of heat stressed tubers on ‘Russet Burbank’ and Li et al. 2017 on postharvest unstressed sprouting tubers of the cultivar Favorita (*R* = 0.323, *P* < 0.01), and highly significant between the Campbell et al. 2014^16^ study on postharvest unstressed tubers of ‘Russet Burbank’ and the Li et al. 2017^17^ study on postharvest unstressed sprouting tubers of ‘Fovarita’ (*R* = 0.476, *P* < 0.01) by regression analysis.
