# Supplementary material for: Heat-stress-induced sprouting and differential gene expression in growing potato tubers: Comparative transcriptomics with that induced by postharvest sprouting
Source: Hortic Res. 2021 Oct 15;8:226. doi: 10.1038/s41438-021-00680-2 (PMC8519922; doi:10.1038/s41438-021-00680-2)
Supplement: Supplementary file 7 — Table S7 [file 41438_2021_680_MOESM7_ESM.docx]

**Table S7. Enriched KEGG pathways in the 1201 differentially expressed genes in the heat-stressed-tuber transcriptome.**

| Pathway | No. of genes | Enrichment factor | *P*-value |
| --- | --- | --- | --- |
| Protein processing in endoplasmic reticulum | 25 | 0.09 | 0.000049 |
| Biosynthesis of secondary metabolites | 76 | 0.06 | 0.000215 |
| Photosynthesis - antenna proteins | 7 | 0.21 | 0.000539 |
| Photosynthesis | 10 | 0.11 | 0.002321 |
| Starch and sucrose metabolism | 14 | 0.09 | 0.002439 |
| Plant-pathogen interaction | 18 | 0.08 | 0.002620 |
| Metabolic pathways | 120 | 0.05 | 0.006765 |
| Carbon fixation in photosynthetic organisms | 8 | 0.10 | 0.013664 |
| Phenylpropanoid biosynthesis | 17 | 0.07 | 0.014961 |
| Diterpenoid biosynthesis | 5 | 0.11 | 0.029152 |
| Pentose phosphate pathway | 6 | 0.10 | 0.031220 |
| Flavonoid biosynthesis | 6 | 0.10 | 0.035372 |
