# Supplementary material for: Heat-stress-induced sprouting and differential gene expression in growing potato tubers: Comparative transcriptomics with that induced by postharvest sprouting
Source: Hortic Res. 2021 Oct 15;8:226. doi: 10.1038/s41438-021-00680-2 (PMC8519922; doi:10.1038/s41438-021-00680-2)
Supplement: Supplementary file 8 — Table S8 [file 41438_2021_680_MOESM8_ESM.docx]

**Table S8. KEGG pathway enrichment analysis results of 88 differentially expressed genes (DEGs) shared between the postharvest sprouting tuber transcriptomes of previous two studies** (**Campbell et al. 2014; Li et al. 2017) and the heat-stressed-tuber transcriptome of this study**

| **Pathway** | **No. genes** | **Enrichment factor** | ***P*-value** |
| --- | --- | --- | --- |
| Photosynthesis - antenna proteins | 2 | 0.06 | 0.005 |
| Diterpenoid biosynthesis | 2 | 0.05 | 0.007 |
| Metabolic pathways | 14 | 0.01 | 0.012 |
| Synthesis and degradation of ketone bodies | 1 | 0.11 | 0.027 |
| Biosynthesis of secondary metabolites | 8 | 0.01 | 0.033 |
| Protein processing in endoplasmic reticulum | 3 | 0.01 | 0.039 |
| AGE-RAGE signaling pathway in diabetic complications | 1 | 0.06 | 0.049 |
