# Supplementary material for: Heat-stress-induced sprouting and differential gene expression in growing potato tubers: Comparative transcriptomics with that induced by postharvest sprouting
Source: Hortic Res. 2021 Oct 15;8:226. doi: 10.1038/s41438-021-00680-2 (PMC8519922; doi:10.1038/s41438-021-00680-2)
Supplement: Supplementary file 9 — Table S9 [file 41438_2021_680_MOESM9_ESM.docx]

**Table S9. Enriched gene ontology (GO) terms of all 1201 DEGs of the heat-stressed-tuber transcriptome**

| **Go term** | **GO ID** | **GO level** | **Genes (n)** | ***P* value** |
| --- | --- | --- | --- | --- |
| MF: Catalytic activity | GO:0003824 | 2 | 571 | 9.02E-04 |
| MF: Molecular transducer activity | GO:0060089 | 2 | 35 | 1.06E-04 |
| CC：Extracellular region | GO:0005576 | 2 | 93 | 3.36E-09 |
| CC：Cell junction | GO:0030054 | 2 | 84 | 4.52E-04 |
| CC：Symplast | GO:0055044 | 2 | 84 | 4.34E-04 |
| BP：Response to stimulus | GO:0050896 | 2 | 370 | 0 |
| BP：Biological regulation | GO:0065007 | 2 | 240 | 2.41E-06 |
| BP：Multicellular organismal process | GO:0032501 | 2 | 171 | 1.11E-08 |
| BP：Developmental process | GO:0032502 | 2 | 163 | 2.41E-06 |
| BP：Multi-organism process | GO:0051704 | 2 | 120 | 1.79E-07 |
| BP：Signaling | GO:0023052 | 2 | 106 | 1.94E-05 |
| BP：Reproduction | GO:0000003 | 2 | 102 | 1.19E-04 |
| BP：Reproductive process | GO:0022414 | 2 | 101 | 1.69E-04 |
| BP：Immune system process | GO:0002376 | 2 | 53 | 5.81E-05 |
