# Supplementary material for: Heat-stress-induced sprouting and differential gene expression in growing potato tubers: Comparative transcriptomics with that induced by postharvest sprouting
Source: Hortic Res. 2021 Oct 15;8:226. doi: 10.1038/s41438-021-00680-2 (PMC8519922; doi:10.1038/s41438-021-00680-2)
Supplement: Supplementary file 10 — Table S10 [file 41438_2021_680_MOESM10_ESM.docx]

**Table S10. Enriched gene ontology terms of 88 DEGs shared between postharvest sprouting tuber transcriptome reported in previous studies (Campbell et al. 2014; Li et al. 2017) and the heat-stressed-tuber transcriptome of our study.**

| **GO term** | **GO ID** | **GO level** | **Enrichment score** | **Genes (n)** | **P value** |
| --- | --- | --- | --- | --- | --- |
| CC：Extracellular region | GO:0005576 | 2 | 3.61 | 14 | 1.60E-05 |
| BP：Reproductive process | GO:0022414 | 2 | 2.66 | 15 | 3.01E-04 |
| BP：Reproduction | GO:0000003 | 2 | 2.65 | 15 | 3.09E-04 |
| BP：Response to stimulus | GO:0050896 | 2 | 1.67 | 31 | 4.28E-04 |
| BP：Multicellular organismal process | GO:0032501 | 2 | 2.10 | 19 | 8.12E-04 |
