# Supplementary material for: Heat-stress-induced sprouting and differential gene expression in growing potato tubers: Comparative transcriptomics with that induced by postharvest sprouting
Source: Hortic Res. 2021 Oct 15;8:226. doi: 10.1038/s41438-021-00680-2 (PMC8519922; doi:10.1038/s41438-021-00680-2)
Supplement: Supplementary file 11 — Table S11 [file 41438_2021_680_MOESM11_ESM.docx]

**Table S11. Distribution of heat-inducible cis-elements (ABRE, G-Box, GC-Motif, and HSE) in the 88 DEGs shared between heat-stressed tuber transcriptome in our study and dormant vs. postharvest sprouting tubers transcriptomes reported previously (Campbell et al. 2014; Li et al. 2017)**

| **DEG ID (PGSC0003+)** | **No. of ABRE** | **No. of G-box** | **No. of GC-motif** | **No. of HSE** | **Sum of these four motifs** | **log_2_ FC in the present study** | **log_2_ FC in Campbell et al. 2014** | **log_2_ FC in Li et al. 2017** | **Function** |
| --- | --- | --- | --- | --- | --- | --- | --- | --- | --- |
| DMT400000089 | 0 | 0 | 0 | 2 | 2 | -3.23 | -1.54 | -1.12 | Acetyl-coenzyme A carboxylase carboxyl transferase alpha |
| DMT400002085 | 0 | 1 | 0 | 0 | 1 | 3.49 | 1.62 | 4.68 | Extensin (ext) |
| DMT400002449 | 6 | 3 | 0 | 0 | 9 | -3.17 | 1.14 | 2.76 | Reticulon family protein |
| DMT400004734 | 0 | 2 | 1 | 1 | 4 | -2.48 | 1.29 | 3.78 | Conserved gene of unknown function |
| DMT400004791 | 3 | 1 | 0 | 1 | 5 | 2.94 | 2.10 | 5.69 | Non-specific lipid-transfer protein 1 |
| DMT400006461 | 5 | 5 | 0 | 0 | 10 | 3.03 | 1.48 | 2.61 | Zinc finger protein |
| DMT400009471 | 1 | 1 | 1 | 0 | 3 | 2.59 | 4.40 | 7.14 | Phenylcoumaran benzylic ether reductase |
| DMT400009481 | 12 | 5 | 0 | 3 | 20 | -2.55 | 1.52 | 1.07 | 50S ribosomal protein L31 |
| DMT400011910 | 4 | 3 | 0 | 1 | 8 | 3.85 | -2.15 | 3.07 | Hydroxyproline-rich glycoprotein (HRGP) |
| DMT400012809 | 4 | 1 | 0 | 0 | 5 | 3.54 | 1.82 | 6.23 | Endo-beta-1,4-glucanase |
| DMT400012829 | 6 | 6 | 0 | 2 | 14 | 3.26 | 1.40 | 3.81 | UDP-glucose 4-epimerase |
| DMT400015740 | 7 | 7 | 0 | 0 | 14 | -4.15 | -1.88 | 1.51 | Chlorophyll a-b binding protein 4, chloroplastic |
| DMT400016545 | 8 | 5 | 0 | 0 | 13 | 2.85 | 1.24 | 7.18 | GDSL-like Lipase/Acylhydrolase family protein |
| DMT400017948 | 3 | 2 | 0 | 3 | 8 | 5.30 | 1.93 | 6.86 | Zinc finger family protein |
| DMT400018296 | 7 | 5 | 0 | 0 | 12 | 3.33 | 1.69 | 2.23 | Epidermis-specific secreted glycoprotein EP1 |
| DMT400019863 | 8 | 12 | 0 | 1 | 21 | -3.45 | 1.80 | 1.48 | Sulfate/bicarbonate/oxalate exchanger and transporter sat-1 |
| DMT400020122 | 2 | 3 | 0 | 1 | 6 | 2.52 | 2.99 | 6.26 | Xyloglucan endotransglucosylase-hydrolase XTH9 |
| DMT400020314 | 6 | 3 | 0 | 1 | 10 | 2.37 | 2.67 | 6.38 | Xyloglucan endotransglucosylase-hydrolase XTH9 |
| DMT400020520 | 0 | 0 | 0 | 2 | 2 | -3.08 | 2.86 | 4.22 | ATP binding protein |
| DMT400021584 | 4 | 3 | 0 | 1 | 8 | 5.47 | 1.66 | 2.29 | Beta-1,3-glucanase, acidic |
| DMT400021662 | 0 | 0 | 0 | 1 | 1 | 3.09 | 2.52 | 2.81 | ATP binding / kinase/ protein serine / threonine kinase |
| DMT400021751 | 1 | 1 | 1 | 1 | 4 | -5.31 | 3.32 | 8.87 | Induced stolon tip protein |
| DMT400023932 | 0 | 0 | 0 | 2 | 2 | -2.85 | -4.30 | 1.45 | Small heat-shock protein homolog protein |
| DMT400023962 | 5 | 1 | 0 | 2 | 8 | -4.27 | 2.09 | 2.89 | Proteinase inhibitor |
| DMT400024338 | 3 | 4 | 0 | 1 | 8 | 3.07 | 2.78 | 7.39 | UDP-glucosyltransferase |
| DMT400024847 | 4 | 2 | 0 | 0 | 6 | 2.41 | 1.36 | 2.72 | Aquaglyceroporin (Tonoplast intrinsic protein (Tipa)) |
| DMT400025955 | 4 | 3 | 0 | 3 | 10 | 2.54 | 2.02 | 6.02 | Patatin 3 |
| DMT400027065 | 0 | 0 | 0 | 1 | 1 | 2.82 | 1.73 | 9.11 | CHP-rich zinc finger protein |
| DMT400028172 | 1 | 2 | 0 | 0 | 3 | 4.44 | 2.15 | 7.68 | Lysine/histidine transporter |
| DMT400028656 | 1 | 1 | 0 | 0 | 2 | 4.71 | 3.70 | 4.21 | UDP-glucuronosyltransferase |
| DMT400029287 | 2 | 2 | 0 | 0 | 4 | -3.86 | 2.09 | 4.01 | P21-rho-binding domain-containing protein |
| DMT400032789 | 2 | 2 | 0 | 0 | 4 | 2.69 | 1.45 | 4.99 | Cationic peroxidase |
| DMT400033248 | 4 | 1 | 1 | 1 | 7 | 3.50 | 1.26 | 3.13 | ESC |
| DMT400033455 | 1 | 4 | 0 | 0 | 5 | 4.82 | 1.91 | 2.07 | Cellulose synthase CslG |
| DMT400035006 | 1 | 1 | 0 | 0 | 2 | -8.89 | -3.17 | 2.04 | Chlorophyll a-b binding protein 3C, chloroplastic |
| DMT400036431 | 5 | 4 | 0 | 0 | 9 | 3.31 | 2.85 | 6.42 | Abhydrolase domain containing |
| DMT400036565 | 4 | 3 | 0 | 0 | 7 | -6.80 | 1.42 | 3.23 | Flavonol synthase |
| DMT400038362 | 2 | 2 | 0 | 2 | 6 | 2.55 | 3.65 | 9.55 | EDGP |
| DMT400041049 | 1 | 1 | 0 | 0 | 2 | 11.48 | 2.93 | 3.37 | Methylketone synthase II |
| DMT400043513 | 6 | 4 | 0 | 1 | 11 | -4.29 | 5.51 | 3.37 | Conserved gene of unknown function |
| DMT400044668 | 5 | 2 | 0 | 0 | 7 | 2.93 | 2.11 | 3.61 | Receptor-like kinase |
| DMT400044708 | 5 | 7 | 0 | 0 | 12 | -4.75 | 2.75 | 8.04 | Conserved gene of unknown function |
| DMT400048685 | 3 | 2 | 0 | 1 | 6 | 3.04 | 3.08 | 9.34 | Polyphenol oxidase A, chloroplastic |
| DMT400048873 | 6 | 4 | 0 | 2 | 12 | 11.70 | 1.94 | 4.35 | Kinesin-3 |
| DMT400049875 | 1 | 2 | 0 | 2 | 5 | -3.39 | 1.81 | 8.72 | Arabinogalactan peptide 20 |
| DMT400053402 | 0 | 0 | 0 | 2 | 2 | -4.29 | -2.04 | 1.66 | Heat-shock protein |
| DMT400054793 | 8 | 6 | 0 | 0 | 14 | 3.76 | 1.64 | 4.22 | BCS1 protein |
| DMT400054947 | 6 | 5 | 0 | 0 | 11 | 2.53 | 1.92 | 3.87 | U-box protein |
| DMT400055203 | 3 | 2 | 1 | 0 | 6 | 3.51 | 2.54 | 5.33 | 1-aminocyclopropane-1-carboxylate synthase 3 |
| DMT400057136 | 4 | 3 | 0 | 2 | 9 | 2.96 | 1.18 | 3.28 | Polygalacturonase inhibitor protein |
| DMT400057397 | 6 | 5 | 0 | 1 | 12 | -4.43 | -2.71 | -1.97 | Conserved gene of unknown function |
| DMT400057515 | 0 | 0 | 0 | 2 | 2 | 3.25 | 1.88 | 4.94 | Gene of unknown function |
| DMT400058561 | 0 | 0 | 0 | 1 | 1 | 3.94 | 1.94 | 2.84 | HMG-CoA synthase |
| DMT400059944 | 5 | 4 | 0 | 0 | 9 | -3.43 | 1.42 | 2.69 | White-brown-complex ABC transporter family |
| DMT400061076 | 4 | 3 | 0 | 0 | 7 | -3.65 | 3.25 | 2.55 | Globulin |
| DMT400061974 | 0 | 1 | 0 | 3 | 4 | 3.28 | 2.18 | 6.13 | Peptide transporter |
| DMT400062314 | 0 | 0 | 0 | 3 | 3 | -4.90 | 3.69 | 1.66 | Gibberellin 20-oxidase-1 |
| DMT400063308 | 3 | 1 | 0 | 0 | 4 | -3.13 | 2.20 | 3.26 | Flavonol 4'-sulfotransferase |
| DMT400063324 | 1 | 1 | 1 | 0 | 3 | -4.43 | -1.16 | -1.39 | Cytochrome P450 |
| DMT400064370 | 7 | 5 | 0 | 0 | 12 | -3.20 | 2.75 | 4.33 | Conserved gene of unknown function |
| DMT400064535 | 4 | 2 | 0 | 1 | 7 | 5.31 | 2.51 | 4.31 | Leucine-rich repeat receptor protein kinase EXS |
| DMT400064581 | 7 | 3 | 1 | 0 | 11 | -2.54 | 2.13 | 1.26 | Conserved gene of unknown function |
| DMT400065197 | 2 | 2 | 0 | 0 | 4 | 4.96 | 1.48 | 3.44 | Nitrate transporter |
| DMT400068267 | 2 | 2 | 0 | 2 | 6 | -2.62 | 1.28 | 2.11 | Conserved gene of unknown function |
| DMT400068332 | 6 | 3 | 0 | 1 | 10 | 4.18 | 3.38 | 4.03 | Transcription factor |
| DMT400069760 | 0 | 1 | 0 | 0 | 1 | -3.20 | -1.41 | -1.11 | Phosphoinositide-specific phospholipase C |
| DMT400069972 | 3 | 1 | 0 | 0 | 4 | 2.67 | 3.59 | 6.03 | Glucosyltransferase |
| DMT400071781 | 4 | 1 | 0 | 0 | 5 | -2.37 | -1.84 | 3.09 | Conserved gene of unknown function |
| DMT400071820 | 1 | 2 | 0 | 2 | 5 | -3.42 | -1.64 | -1.13 | ER lumen retaining receptor family |
| DMT400073355 | 6 | 2 | 0 | 0 | 8 | -4.50 | -1.92 | -1.17 | SNF4 |
| DMT400075057 | 9 | 8 | 0 | 2 | 19 | -4.92 | -2.39 | -1.03 | Ferritin |
| DMT400076784 | 4 | 3 | 0 | 1 | 8 | -6.16 | 2.59 | 4.26 | Conserved gene of unknown function |
| DMT400078006 | 4 | 2 | 0 | 3 | 9 | -4.46 | -2.41 | 1.23 | 17.6 kD class I small heat shock protein |
| DMT400078609 | 1 | 1 | 0 | 0 | 2 | 2.86 | 1.76 | 8.41 | Proteinase inhibitor IIa |
| DMT400079203 | 0 | 1 | 0 | 0 | 1 | 2.95 | 2.58 | 7.12 | Glycosyltransferase |
| DMT400080959 | 5 | 3 | 0 | 0 | 8 | 2.50 | 5.43 | 5.43 | Glycine-rich cell wall structural protein |
| DMT400081244 | 4 | 3 | 0 | 0 | 7 | 2.79 | -1.99 | 8.72 | Phospholipase A1 |
| DMT400081314 | 3 | 2 | 0 | 0 | 5 | 3.26 | 1.25 | 3.66 | Endo-1,4-beta-glucanase |
| DMT400083791 | 0 | 1 | 0 | 0 | 1 | 11.70 | -2.20 | 4.63 | Periaxin |
| Total | 3.35 | 2.56 | 0.09 | 0.82 | 6.82 | 0.61 | 1.39 | 3.98 |  |

# Common DEGs shared by our study on heat stressed tubers and previous *studies*^16, 17^on postharvest sprouting tubers*.*
