# Supplementary material for: Heat-stress-induced sprouting and differential gene expression in growing potato tubers: Comparative transcriptomics with that induced by postharvest sprouting
Source: Hortic Res. 2021 Oct 15;8:226. doi: 10.1038/s41438-021-00680-2 (PMC8519922; doi:10.1038/s41438-021-00680-2)
Supplement: Supplementary file 12 — Figure S1 [file 41438_2021_680_MOESM12_ESM.docx]

**
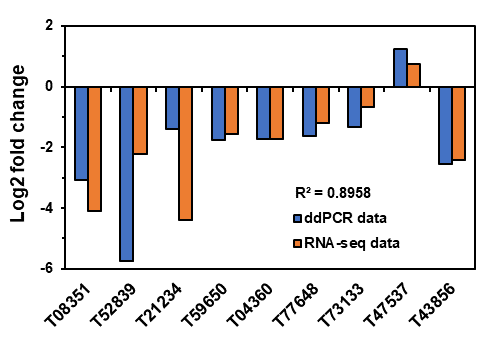
**

**Fig. S1. Validation of the RNA sequencing data using RT-ddPCR. ‘**T’ in name of nine “cDNA genes” represent the abbreviation of PGSC0003DMT4000. ddPCR: absolute quantification using reverse transcriptase digital droplet PCR. The log_2_ fold change values of gene expression were for HS tubers/CK tubers. *R^2^* = 0.8958 and *P* < 0.001 were from the log_­­2_ value regression analysis between the RNA-seq analysis and the ddPCR analysis.
